# Association of Dietary Carrot/Carotene Intakes With Colorectal Cancer Incidence and Mortality in the Prostate, Lung, Colorectal, and Ovarian Cancer Screening Trial

**DOI:** 10.3389/fnut.2022.888898

**Published:** 2022-06-17

**Authors:** Zongze Jiang, Huilin Chen, Ming Li, Wei Wang, Chuanwen Fan, Feiwu Long

**Affiliations:** ^1^Department of Gastrointestinal Surgery, Bariatric and Metabolic Surgery, West China School of Public Health and West China Fourth Hospital, Sichuan University, Chengdu, China; ^2^Research Center for Nutrition, Metabolism and Food Safety, West China-PUMC C.C. Chen Institute of Health, Sichuan University, Chengdu, China; ^3^Department of Immunology, Institute of Basic Medical Sciences, Chinese Academy of Medical Sciences, Beijing, China; ^4^School of Basic Medicine, Peking Union Medical College, Beijing, China; ^5^Department of Nutrition, Food Hygiene and Toxicology, Healthy Food Evaluation Research Center, West China School of Public Health and West China Fourth Hospital, Sichuan University, Chengdu, China

**Keywords:** carrot, carotene, colorectal cancer, cohort, PLCO

## Abstract

**Background:**

The evidence of dietary carrot/carotene intake's effect on the association with colorectal cancer (CRC) risk is conflicted. We sought to examine the association of carrot/carotene intake with CRC incidence and mortality in the Prostate, Lung, Colorectal, and Ovarian Cancer (PLCO) Screening cohort.

**Methods:**

In all, 101,680 participants were enrolled between November 1993 and July 2001 from the PLCO cohort. We employed the multivariable Cox regression analyses to estimate the hazard ratios and 95% confidence interval. Subgroup analyses and interaction tests were performed to examine the potential effect modifiers. We further applied the generalized additive model to explore the non-linear trend of the exposure to cancer-related outcomes.

**Results:**

A total of 1,100 CRC cases and 443 cancer-related deaths were documented. We noted that the 4th quintile of dietary carrot intakes was associated with a 21% lower risk of CRC incidence, compared with the lowest quintile group (full-adjusted HR_quintile4vs.quintile1_ = 0.79, 95%CI = 0.65–0.97, *p* for trend = 0.05), while the adjusted-HR was 0.95 (95%CI = 0.89–1.02) with per SD increment of carrot intakes, and no statistically significant associations were detected between dietary α-, and β-carotene intake and CRC incidence. There were no statistically significant associations observed between carrot/carotene intakes and CRC mortality. Furthermore, there were no non-linear dose-response relationships between dietary carrot, α-, and β-carotene intake and CRC incidence and mortality (all *p*_nonlinearity_ > 0.05). Of note, smoking status as a modifier on the association of dietary carrot intakes with CRC incidence but not mortality was observed.

**Conclusions:**

In summary, this large U.S. prospective cohort study indicated that a moderate consumption of carrots was associated with a lower CRC incidence, which suggested that a certain dose-range of carrots consumed might contribute to a potential cancer-prevention effect, not the more the better.

## Introduction

Colorectal cancer (CRC) is the third most common cause of cancer-related death in the United States, with nearly 147,950 incident cases and 53,200 cancer deaths in 2020 (1). Apart from well-established risk factors (i.e., environmental and genetic factors) that play a crucial role in the pathogenesis of CRC (2, 3), more than half of patients can be attributed to other risk factors including smoking, diet, drinking, obesity, and thus may be potentially preventable (4). Although emerging evidence implies that cancer prevention dietary nutrients or food, including calcium (5, 6), fiber (7), dairy products (8), and whole grain (9) have been associated with a lower risk of colorectal cancer, it remains controversial.

Carrots are rich in high amounts of carotenoid antioxidants (α- and -β-carotene) that might have a potential role in cancer prevention (10, 11). Several meta-analyses on carrot consumption have indicated that carrot intake was inversely associated with the risk of several cancers, including gastric (12), lung (13), prostate (14), breast (15), and urothelial cancer (16). However, epidemiological studies depicted an inverse (17–20) or a null association (21–23) of dietary α-/β-carotene intakes with the risk of CRC. Recently, a prospective cohort study of 57,053 Danes has shown that the consumption of raw carrots was associated with a 17% decrease in the risk of CRC (24). Given the differences in geography and eating habits, it remains unclear whether the results would be stable for U.S. adults. Meanwhile, there is no evidence of the effect of dietary carrot intake on mortality of CRC.

Therefore, to provide the most reliable prospective evidence on the association of dietary carrot intake with the risk of CRC, we, respectively, analyze the association of dietary carrot, α- and -β-carotene intakes with the risk of CRC incidence and mortality using a multicenter randomized controlled trial data from the prostate, lung, colorectal, and ovarian (PLCO) screening trial.

## Methods

### Data Source and Study Population

The PLCO Cancer Screening Trial is a randomized, controlled trial conducted to investigate whether certain screening examinations would reduce the mortality from PLCO cancers. The specific study design and methods were previously illustrated elsewhere (25). Approximately 155,000 participants were recruited from 1993 to 2001 via 10 screening centers across the United States, through a detailed recruitment plan. Then, they were randomly assigned to two groups (the control group receiving usual care, whereas the intervention arm receiving screening tests). The PLCO study was approved by the Institutional Review Boards of the US National Cancer Institute and each study center, and written informed consents were obtained from all eligible participants.

We established inclusion criteria to identify eligible cases in our final cohort. They would be further excluded as follows: (1) no baseline questionnaire returned (BQ) (*n* = 4,918) and any history of colorectal cancer before BQ (*n* = 34); (2) did not complete Diet History Questionnaire (DHQ) (*n* = 33,230), and invalid DHQ for missing the date of DHQ completion, the date of DHQ completion before the date of death, the presence of ≥8 missing frequency responses or extreme values of calorie intake [i.e., top 1% or bottom 1%]) (*n* = 5,221); (3) a history of any cancer before DHQ entry (*n* = 9,682), and no follow-up time after the DHQ (*n* = 122). At last, 101,680 subjects were included in our cohort.

### Data Collection and Dietary Assessment

The self-reported information of sex, race, trial arm, body mass index (BMI), educational level, marital status, aspirin use, cigarette smoking, family history of colorectal cancer, and diabetes history, were collected from the BQ. Dietary data, including age at DHQ, alcohol drinking, dietary carrot/carotene intake, energy intake from diet, and supplemental nutrients (Beta-Carotene, Calcium, Vitamin A, Vitamin C, Vitamin D, and Vitamin E), were collected from the DHQ (version 1.0, National Cancer Institute 2007), which was a self-administered food frequency questionnaire (FFQ) designed to assess the portion size and consumption frequency of 124 food items and supplement use over the past year, and has been validated with better performance in estimating dietary intake than two widely used FFQs (26) at the time of PLCO study carried out. The 1994–96 Continuing Survey of Food Intakes by Individuals, available from the USDA Food Surveys Research Group, and the Nutrition Data Systems for Research (NDS-R) from the University of Minnesota was applied to calculate the daily intake of all nutrients in the database (27). Three exposure variables (dietary carrot, α-, and dietary β-carotene intakes) were acquired in this study.

### Ascertainment of Colorectal Cancer Incidence and Mortality

The time metric was followed up from the date of DHQ completion to the date of events that firstly occurred, including colorectal cancer diagnosis, dropout, colorectal cancer death, or the end of follow-up (incidence through December 31, 2009; and mortality through December 31, 2015). CRC diagnosis was ascertained via an annually updated medical record. Deaths were mainly identified by (1) annually mailed questionnaires, (2) reports from relatives, friends, or physicians, and (3) periodic linkage to the National Death Index. Our interested endpoints were the incidence and mortality of colorectal cancer.

### Statistical Analysis

Dietary carrot, α-, and β-carotene intakes were adjusted for energy intake with the residual method (28). The distributions of them were transformed with Z-score as continuous variables, and then were divided into quintiles as categorical variables, and the lowest quintile was as the referent. Continuous variables are expressed as median (IQR, interquartile range), and categorical variables are presented as numbers (frequency). We applied the Kruskal–Wallis H test and Pearson's chi-squared test to compare the baseline differences, if appropriate. Multivariable Cox regression analyses were employed to estimate the hazard ratios (HR) and 95% confidence interval (CI). The proportional hazard assumption of baseline covariates for the Cox model was verified using the Schoenfeld residuals (29) (all *p* > 0.05). Potential confounders were selected according to the change-in-estimate strategy (30) (more than 10% change in effect estimates) and literature-known risk factors. Missing values of covariates were treated as dummy variables in the multivariable Cox regression analyses. Specifically, the full-adjusted model included age, sex, race, trial arm, marital status, BMI, educational level, aspirin use, cigarette smoking, alcohol drinking, diabetes, family history of colorectal cancer, and energy intake from the diet, and supplemental use of Beta-Carotene, Calcium, Vitamin A, Vitamin C, Vitamin D, and Vitamin E. We also analyzed the linear trend of each quintile of energy-adjusted dietary carrot, α-, and β-carotene intakes, by entering the median value as a continuous variable in the model.

Subgroup analyses were performed, including age, sex, race, trial arm, marital status, BMI, educational level, aspirin use, cigarette smoking, alcohol drinking, diabetes, and family history of colorectal cancer. The interaction effect on each stratum was compared using likelihood-ratio tests. To address the dose-response trend between dietary carrot/carotene intakes and colorectal cancer incidence and mortality, the smooth curve fitting was conducted with a multivariable Cox regression model using the generalized additive model (GAM, Restricted Cubic Spline Functions). Here, we excluded subjects with <1st or >90th percentile values of energy-adjusted dietary carrot/carotene intakes (i.e., to reduce the potential impacts on the association of dose-response analyses).

The following sensitivity analyses were conducted: (1) excluding cases diagnosed or died within the first 5 years of follow-up; (2) excluding the extreme values of energy intake from the diet (<800/>4,000 kcal/day for men and <500/>3,500 kcal/day for women); (3) additional adjusted for other factors, including fruit (continuous), dietary Magnesium (continuous), dietary Sodium (continuous), dietary Potassium (continuous), whole grain (continuous), vegetables (continuous), added sugars (continuous), fiber (continuous), and saturated fatty acids (continuous). All analyses were performed using R Statistical Software (http://www.R-project.org, The R Foundation) and the Free Statistics analysis platform (31). Tests were two-tailed, and the significance level was set at 0.05.

## Results

### Participant Characteristics

**Figure 1:**
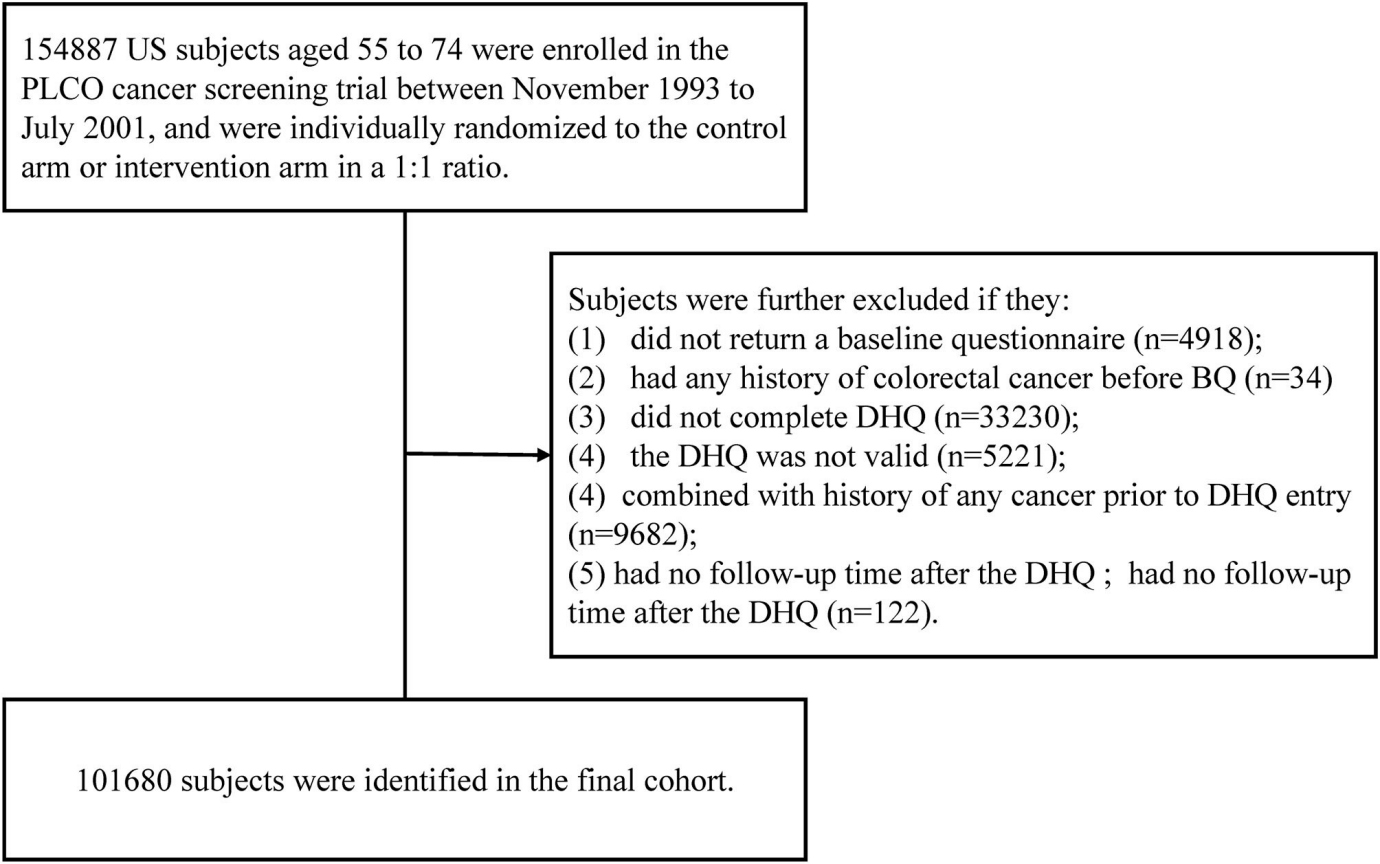
The flow chart of study participants from the PLCO screening trial.

Accordingly, 101,680 subjects were selected for the final data analysis (Figure 1). The baseline characteristics are presented in Table 1. The median value of dietary carrot intake (without adjustment for energy intake) was 4.3 g/day, ranging from 0 to 205.3 g/day. Compared to participants with the highest quintile (Q5) of dietary carrot consumption, participants with the lowest quintile (Q1) showed a higher proportion of males (72.8% vs. 36.2%), Black race, aspirin usage, higher BMI (70.4% vs. 60.4%), and higher energy intake (2132.0 kcal/day vs. 1597.0 kcal/day), and former/current cigarette smoking (48.2% vs. 39.3%;15.4% vs. 5.0%).

**Table 1:** Baseline characteristics of study population according to quintiles of energy-adjusted dietary carrot intake in 101680 US participants.

		**Quintiles of energy-adjusted dietary carrot intake, g/day**				
**Variables^a^**	**Overall**	**Q1 (<1.95)**	**Q2 (1.95–3.88)**	**Q3 (3.88–7.60)**	**Q4 (7.60–15.30)**	**Q5 (>15.30)**
Number of participants	101,680	20,336	20,336	20,336	20,336	20,336
Age at DHQ (years)	65.0 (61.0, 70.0)	64.0 (60.0, 69.0)	65.0 (61.0, 70.0)	66.0 (61.0, 70.0)	66.0 (61.0, 70.0)	65.0 (61.0, 70.0)
**Sex**						
Male	49,441 (48.6)	14,802 (72.8)	10,366 (51)	8,618 (42.4)	8,299 (40.8)	7,356 (36.2)
Female	52,239 (51.4)	5,534 (27.2)	9,970 (49)	11,718 (57.6)	12,037 (59.2)	12,980 (63.8)
**Trial arm**						
Intervention	51,767 (50.9)	10,432 (51.3)	10,362 (51)	10,308 (50.7)	10,384 (51.1)	10,281 (50.6)
Control	49,913 (49.1)	9,904 (48.7)	9,974 (49)	10,028 (49.3)	9,952 (48.9)	10,055 (49.4)
**Race**						
White, Non-Hispanic	92,465 (90.9)	17,726 (87.2)	17,982 (88.4)	18,563 (91.3)	19,034 (93.6)	19,160 (94.2)
Black, Non-Hispanic	3,352 (3.3)	1,269 (6.2)	947 (4.7)	521 (2.6)	322 (1.6)	293 (1.4)
Hispanic	1,493 (1.5)	418 (2.1)	342 (1.7)	272 (1.3)	215 (1.1)	246 (1.2)
Others^b^	4,333 (4.3)	912 (4.5)	1,055 (5.2)	974 (4.8)	759 (3.7)	633 (3.1)
Missing	37 (0.0)	11 (0.1)	10 (0)	6 (0)	6 (0)	4 (0)
**Marital status**						
Married	79,578 (78.3)	15,647 (76.9)	15,742 (77.4)	15,851 (77.9)	16,351 (80.4)	15,987 (78.6)
Unmarried	21,916 (21.6)	4,648 (22.9)	4,553 (22.4)	4,445 (21.9)	3,951 (19.4)	4,319 (21.2)
Missing	186 (0.2)	41 (0.2)	41 (0.2)	40 (0.2)	34 (0.2)	30 (0.1)
**Education level**						
College below	64,704 (63.6)	13,517 (66.5)	13,500 (66.4)	13,408 (65.9)	12,499 (61.5)	11,780 (57.9)
College graduate	17,838 (17.5)	3,466 (17)	3,372 (16.6)	3,385 (16.6)	3,724 (18.3)	3,891 (19.1)
Postgraduate	18,941 (18.6)	3,314 (16.3)	3,418 (16.8)	3,500 (17.2)	4,080 (20.1)	4,629 (22.8)
Missing	197 (0.2)	39 (0.2)	46 (0.2)	43 (0.2)	33 (0.2)	36 (0.2)
**Aspirin use**						
No	53,472 (52.6)	10,082 (49.6)	10,763 (52.9)	10,849 (53.3)	10,789 (53.1)	10,989 (54)
Yes	47,775 (47.0)	10,148 (49.9)	9,475 (46.6)	9,401 (46.2)	9,467 (46.6)	9,284 (45.7)
Missing	433 (0.4)	106 (0.5)	98 (0.5)	86 (0.4)	80 (0.4)	63 (0.3)
**Diabetes**						
No	94,353 (92.8)	18,702 (92)	18,695 (91.9)	18,867 (92.8)	19,002 (93.4)	19,087 (93.9)
Yes	6,801 (6.7)	1,536 (7.6)	1,525 (7.5)	1,353 (6.7)	1,241 (6.1)	1,146 (5.6)
Missing	526 (0.5)	98 (0.5)	116 (0.6)	116 (0.6)	93 (0.5)	103 (0.5)
**Cigarette smoking**						
Never	48,532 (47.7)	7,396 (36.4)	9,063 (44.6)	10,071 (49.5)	10,674 (52.5)	11,328 (55.7)
Current	9,393 (9.2)	3,132 (15.4)	2,151 (10.6)	1,768 (8.7)	1,322 (6.5)	1,020 (5)
Former	43,742 (43.0)	9,805 (48.2)	9,120 (44.8)	8,493 (41.8)	8,337 (41)	7,987 (39.3)
Missing	13 (0.0)	3 (0)	2 (0)	4 (0)	3 (0)	1 (0)
**BMI, kg/m** ^**2**^						
<25	34,426 (33.9)	5,729 (28.2)	6,550 (32.2)	7,066 (34.7)	7,269 (35.7)	7,812 (38.4)
≤ 25	65,915 (64.8)	14,318 (70.4)	13,507 (66.4)	12,988 (63.9)	12,823 (63.1)	12,279 (60.4)
Missing	1,339 (1.3)	289 (1.4)	279 (1.4)	282 (1.4)	244 (1.2)	245 (1.2)
**Family history of colorectal cancer**						
No	88,113 (86.7)	17,659 (86.8)	17,603 (86.6)	17,649 (86.8)	17,668 (86.9)	17,534 (86.2)
Yes	10,300 (10.1)	1,894 (9.3)	2,014 (9.9)	2,039 (10)	2,127 (10.5)	2,226 (10.9)
Possibly	2,493 (2.5)	625 (3.1)	554 (2.7)	495 (2.4)	408 (2)	411 (2)
Missing	774 (0.8)	158 (0.8)	165 (0.8)	153 (0.8)	133 (0.7)	165 (0.8)
Energy intake from diet, kcal/day	1607.0 (1222.0, 2101.0)	2132.0 (1727.0, 2668.0)	1372.0 (1083.0, 1721.0)	1300.0 (991.6, 1868.0)	1610.0 (1299.0, 1985.0)	1597.0 (1236.0, 2118.0)
**Alcohol drinking**						
Never	10,110 (9.9)	1,370 (6.7)	1,941 (9.5)	2,153 (10.6)	2,188 (10.8)	2,458 (12.1)
Former	14,746 (14.5)	3,304 (16.2)	3,088 (15.2)	2,991 (14.7)	2,656 (13.1)	2,707 (13.3)
Current	73,944 (72.7)	15,125 (74.4)	14,707 (72.3)	14,569 (71.6)	14,943 (73.5)	14,600 (71.8)
Missing	2,880 (2.8)	537 (2.6)	600 (3)	623 (3.1)	549 (2.7)	571 (2.8)
Supplemental Beta-Carotene, mcg/day	142.9 (0.0, 200.0)	57.1 (0.0, 200.0)	142.9 (0.0, 200.0)	142.9 (0.0, 200.0)	200.0 (0.0, 200.0)	200.0 (0.0, 200.0)
Supplemental Vitamin A, i.u./day	3571.0 (0.0, 5000.0)	1429.0 (0.0, 5000.0)	3571.0 (0.0, 5000.0)	3571.0 (0.0, 5000.0)	5000.0 (0.0, 5000.0)	5000.0 (0.0, 5000.0)
Supplemental Vitamin E, mg/day	20.1 (0.0, 288.1)	20.1 (0.0, 268.0)	20.1 (0.0, 268.0)	20.1 (0.0, 288.1)	50.2 (5.7, 288.1)	115.8 (14.4, 288.1)
Supplemental Vitamin C, mg/day	60.0 (0.0, 400.0)	60.0 (0.0, 310.0)	60.0 (0.0, 310.0)	60.0 (0.0, 400.0)	60.0 (1.0, 500.0)	60.0 (17.1, 500.0)
Supplemental Calcium, mg/day	4.1 (0.0, 500.0)	0.0 (0.0, 171.4)	0.0 (0.0, 500.0)	16.4 (0.0, 500.0)	33.2 (0.0, 600.0)	142.9 (0.0, 600.0)

a *Data are presented as median (IQR) or number (percentage)*.

b *“Others” refers to Asian, Pacific Islander, or American Indian. DHQ, dietary history of questionnaire; BMI, body mass index*.

### Energy-Adjusted Dietary Carrot/Carotene Intakes and Colorectal Cancer Incidence

A total of 1,100 participants were diagnosed with CRC after a median follow-up of 9.4 years (896,327.8 person-years), and the incidence rate was 12.27 per 10,000 person-years. As shown in Table 2, we observed an inverse effect on the association between carrot intakes and CRC incidence at the quintile 4 level, which showed the multivariable-adjusted HR was 0.79 (95%CI = 0.65–0.97, *p*_trend_ = 0.05), compared with the referent group; and corresponding adjusted HR of cancer risk was 0.95 (95%CI = 0.89–1.02) with per SD increment of carrot intakes. However, no statistical association was detected between dietary α- and β-carotene intakes and CRC incidence after adjusting for covariates.

**Table 2:** Association between energy-adjusted dietary carrot/carotene intakes and colorectal cancer incidence risk in the PLCO cancer screening trial.

					**HR (95%CI)**, ***p*****-value**			
**Variable**	**Cohort (*n*)**	**Cases (*n*)**	**Person-years**	**Incidence rate per** **10,000 person-years**	**Unadjusted**	**Model 1**	**Model 2**	**Model 3**
**Dietary carrot intakes, g/day**								
Q1 (<1.95)	20,336	239	175,553.87	13.61	1 (Ref)	1 (Ref)	1 (Ref)	1 (Ref)
Q2 (1.95–3.88)	20,336	240	177,803.05	13.50	0.99 (0.83–1.19), *p =* 0.928	0.99 (0.82–1.18), *p =* 0.874	1 (0.82–1.21), *p =* 0.997	1 (0.82–1.21), *p =* 0.995
Q3 (3.88–7.60)	20,336	235	179,671.94	13.08	0.96 (0.8–1.15), *p =* 0.668	0.96 (0.8–1.16), *p =* 0.701	0.99 (0.82–1.2), *p =* 0.922	1 (0.82–1.21), *p =* 0.968
Q4 (7.60–15.30)	20,336	185	181,363.20	10.20	0.75 (0.62–0.91), *p =* 0.003	0.75 (0.62–0.91), *p =* 0.004	0.79 (0.64–0.96), *p =* 0.019	0.79 (0.65–0.97), *p =* 0.025
Q5 (>15.30)	20,336	201	181,935.76	11.05	0.81 (0.67–0.98), *p =* 0.03	0.83 (0.69–1.01), *p =* 0.062	0.88 (0.73–1.08), *p =* 0.225	0.9 (0.74–1.1), *p =* 0.306
*T*rend					0.001	0.004	0.029	0.05
Per SD increment	101,680	1,100	896,327.81	12.27	0.91 (0.85–0.98), *p =* 0.011	0.93 (0.87–0.99), *p =* 0.034	0.95 (0.88–1.01), *p =* 0.117	0.95 (0.89–1.02), *p =* 0.169
**Dietary** **α-carotene intakes, mcg/day**								
Q1 (<317.9)	20,336	236	176,237.15	13.39	1 (Ref)	1 (Ref)	1 (Ref)	1 (Ref)
Q2 (317.9–475.86)	20,336	238	178,171.74	13.36	1 (0.83–1.19), *p =* 0.981	1 (0.83–1.19), *p =* 0.961	1.02 (0.84–1.24), *p =* 0.84	1.02 (0.84–1.24), *p =* 0.82
Q3 (475.86–697.34)	20,336	233	179,532.58	12.98	0.97 (0.81–1.16), *p =* 0.74	0.97 (0.8–1.16), *p =* 0.717	1.01 (0.83–1.23), *p =* 0.928	1.01 (0.83–1.23), *p =* 0.892
Q4 (697.35–1135.29)	20,336	188	180,743.42	10.40	0.78 (0.64–0.94), *p =* 0.01	0.77 (0.63–0.94), *p =* 0.009	0.82 (0.67–1.02), *p =* 0.076	0.83 (0.67–1.03), *p =* 0.087
Q5 (>1135.37)	20,336	205	181,642.92	11.29	0.84 (0.7–1.02), *p =* 0.075	0.85 (0.7–1.03), *p =* 0.107	0.96 (0.75–1.24), *p =* 0.773	0.97 (0.76–1.25), *p =* 0.832
*T*rend					0.006	0.01	0.217	0.249
Per SD increment	101680	1,100	896327.81	12.27	0.93 (0.87–0.99), *p =* 0.027	0.94 (0.88–1), *p =* 0.059	0.99 (0.89–1.1), *p =* 0.814	0.99 (0.89–1.1), *p =* 0.833
**Dietary** **β-carotene intakes, mcg/day**								
Q1 (<1707.28)	20,336	238	176,127.95	13.51	1 (Ref)	1 (Ref)	1 (Ref)	1 (Ref)
Q2 (1707.28–2389.73)	20,336	250	177,981.12	14.05	1.04 (0.87–1.24), *p =* 0.668	1.04 (0.87–1.25), *p =* 0.651	1.06 (0.88–1.28), *p =* 0.513	1.07 (0.89–1.29), *p =* 0.477
Q3 (2389.74–3239.34)	20,336	199	180,083.18	11.05	0.82 (0.68–0.99), *p =* 0.037	0.83 (0.69–1.01), *p =* 0.062	0.86 (0.7–1.05), *p =* 0.147	0.87 (0.71–1.07), *p =* 0.177
Q4 (3239.4–4920.97)	20,336	227	180,603.16	12.57	0.93 (0.78–1.12), *p =* 0.44	0.95 (0.79–1.15), *p =* 0.59	1 (0.81–1.23), *p =* 0.991	1.01 (0.83–1.24), *p =* 0.893
Q5 (>4921.39)	20,336	186	181,532.41	10.25	0.76 (0.63–0.92), *p =* 0.005	0.78 (0.64–0.95), *p =* 0.015	0.82 (0.63–1.07), *p =* 0.151	0.84 (0.64–1.1), *p* = 0.197
*T*rend					0.002	0.009	0.181	0.243
Per SD increment	101,680	1,100	896327.8137	12.27	0.92 (0.86–0.98), *p =* 0.013	0.93 (0.87–1), *p =* 0.036	0.96 (0.86–1.07), *p =* 0.44	0.97 (0.87–1.07), *p =* 0.519

*PLCO, prostate, lung, colorectal, and ovarian; HR, hazard ratio; CI, confidence interval; SD, standard deviation*.

*Unadjusted was the crude model.*

*Model 1 adjusted for age (continuous) and sex (male vs. female)*.

*Model 2 adjusted for model 1 plus trial arm (intervention vs. control), race (white, non-Hispanic vs. black, non-Hispanic vs. Hispanic vs. others), marital status (married vs. unmarried), education level (college below vs. college graduate vs. postgraduate), aspirin use (yes vs. no), diabetes (yes vs. no), cigarette smoking (never vs. current vs. former), BMI (<25 vs. ≤ 25 kg/m^2^),family history of colorectal cancer (yes vs. no vs. possibly), energy intake from diet (continuous), alcohol drinking (never vs. former vs. current), supplemental Beta-Carotene (continuous)*.

*Model 3 adjusted for model 2 plus supplemental vitamin A (continuous), supplemental vitamin E (continuous), supplemental vitamin C (continuous), supplemental calcium (continuous)*.

*For the association of dietary α-carotene intake with colorectal cancer incidence, model 2 was further adjusted for energy-adjusted dietary β-carotene intake (mcg/day). For the association of dietary β-carotene intake with colorectal cancer incidence, model 2 was further adjusted for energy-adjusted dietary α-carotene intake (mcg/day)*.

**Figure 2:**
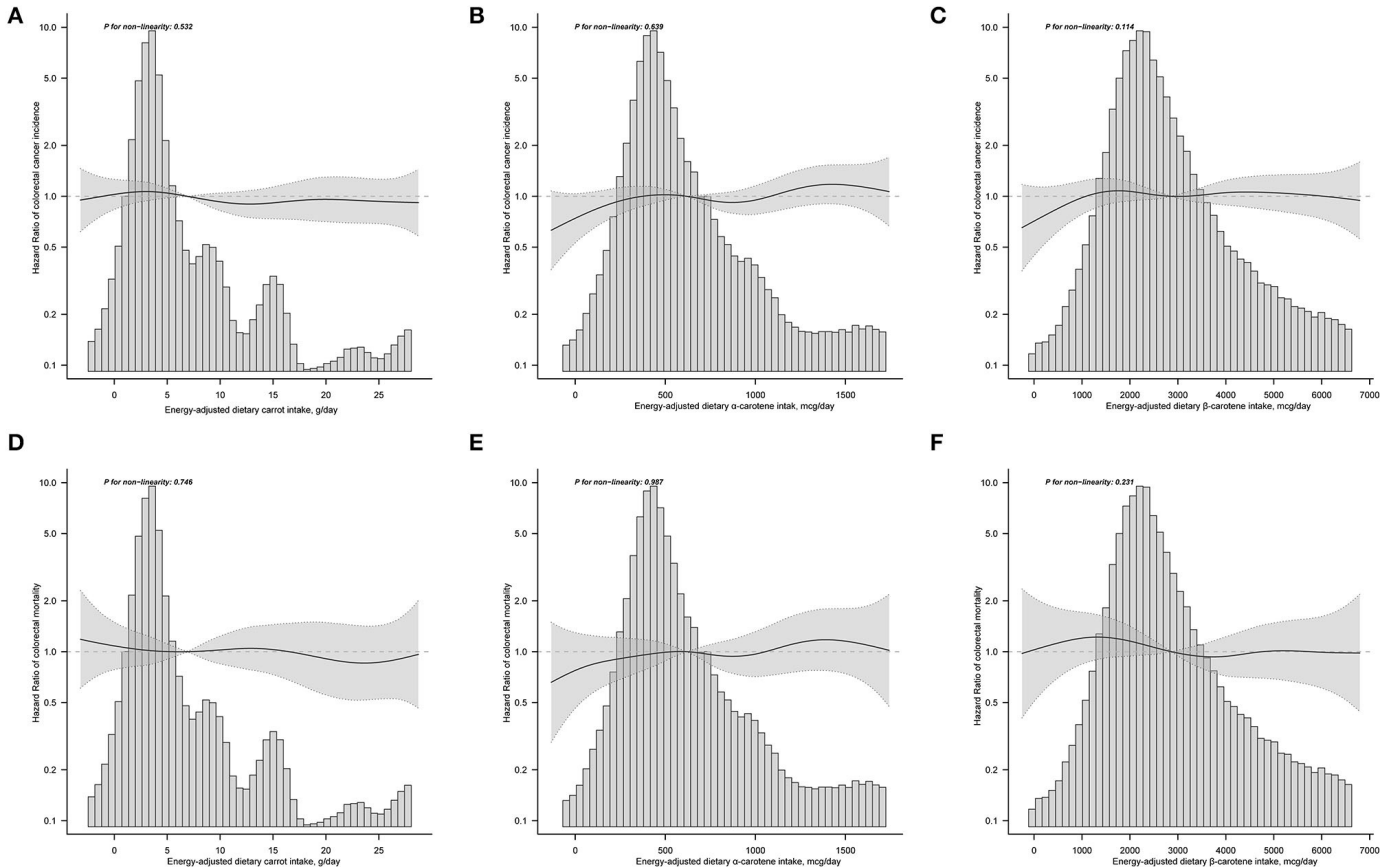
Does-response analyses for the associations between energy-adjusted dietary carrot intake **(A)**, dietary α-carotene intake **(B)**, dietary β-carotene intake **(C)** and colorectal cancer incidence; and the associations between energy-adjusted dietary carrot intake **(D)**, dietary α-carotene intake **(E)**, dietary β-carotene intake **(F)** and colorectal cancer mortality were performed with smooth curve fitting using the generalized additive model. Hazard ratios and 95% confidence interval were calculated by the fully-adjusted multivariable Cox regression model, including age, sex, race, trial arm, marital status, BMI, educational level, aspirin use, cigarette smoking, alcohol drinking, diabetes, family history of colorectal cancer, energy intake from diet, supplemental Beta-Carotene, supplemental Calcium, supplemental Vitamin A, supplemental Vitamin C, supplemental Vitamin D, and Supplemental Vitamin E. Solid lines represent point estimates and dashed lines represent corresponding 95% confidence intervals. The histograms show the percentage of participants belonging to each level of specific energy-adjusted dietary carrot/carotene intake.

The results of subgroup analyses indicated that the associations between dietary carrot intakes and the risk of CRC were stable in almost all subgroups (Supplementary Table 1). An exception of significant effect modifier was represented by smoking status, which showed a stronger association for the participants who never smoked than those with a history of smoking (*p* for interaction = 0.027). Sensitivity analyses revealed that the results of the correlation between carrot/carotene intake and CRC risk were substantially robust to the findings in Table 2 and Supplementary Table 3. The smooth curve fitting plots of carrot/carotene intakes with CRC risk revealed no evidence of a non-linear trend (all *p* for non-linearity > 0.05, Figures 2A–C).

### Energy-Adjusted Dietary Carrot/Carotene Intakes and Colorectal Cancer Mortality

A total of 443 cases died from CRC after a median follow-up of 14.5 years (1,353,326.28 person-years), and the mortality rate was 3.27 per 10,000 person-years. As shown in Table 3, in the fully-adjusted Cox model, only a suggestive but no significant association with cancer mortality was noted for carrot intakes (HR_quintile5vs.quintile1_ = 0.87, 95%CI = 0.64–1.18, *p*_trend_ = 0.297), and corresponding adjusted HR was 0.94 (95%CI = 0.84–1.05), with per SD increment of carrot intakes. Similar results on the association of dietary α-carotene intake with colorectal mortality were obtained (model 3: HR_quintile5vs.quintile1_ = 0.91, 95%CI = 0.62–1.35, *p*_trend_ = 0.417; and HR with per SD increment = 0.94, 95%CI = 0.8–1.11); and for dietary β-carotene intake (model 3: HR quintile 5 *vs*. quintile 1 = 0.91, 95%CI = 0.61–1.36, *p* trend = 0.344; and HR with per SD increment = 1.00, 95%CI = 0.86–1.17). Results of subgroup analyses showed no significant effect modifies in the prespecified groups (all *p* for interaction > 0.05, Supplementary Table 2). In the sensitivity analyses, the null associations of dietary carrot/carotene intakes were robust to colorectal cancer mortality (Supplementary Table 4). Dose-response analyses suggested no non-linear relationship between carrot/carotene intakes and colorectal cancer mortality (all *p* for non-linearity > 0.05, Figures 2D–F).

**Table 3:** Association between energy-adjusted dietary carrot/carotene intakes and colorectal cancer mortality risk in the PLCO cancer screening trial.

					**HR (95%CI)**, ***P*****-value**			
**Variable**	**Cohort (*n*)**	**Cases (*n*)**	**Person-years**	**Mortality rate per** **10,000 person-years**	**Unadjusted**	**Model 1**	**Model 2**	**Model 3**
**Dietary carrot intakes, g/day**								
Q1 (<1.95)	20,336	103	264,449.34	3.90	1 (Ref)	1 (Ref)	1 (Ref)	1 (Ref)
Q2 (1.95–3.88)	20,336	92	267,822.85	3.44	0.88 (0.66–1.17), *p =* 0.374	0.88 (0.66–1.17), *p =* 0.377	0.94 (0.69–1.28), *p =* 0.695	0.94 (0.7–1.28), *p =* 0.71
Q3 (3.88–7.60)	20,336	87	270,611.78	3.22	0.82 (0.62–1.09), *p =* 0.178	0.83 (0.62–1.12), *p =* 0.223	0.9 (0.67–1.23), *p =* 0.522	0.91 (0.67–1.24), *p =* 0.569
Q4 (7.60–15.30)	20,336	82	274,063.17	2.99	0.76 (0.57–1.02), *p =* 0.067	0.77 (0.57–1.03), *p =* 0.082	0.85 (0.63–1.15), *p =* 0.296	0.87 (0.64–1.18), *p =* 0.359
Q5 (>15.30)	20,336	79	276,379.15	2.86	0.73 (0.54–0.97), *p =* 0.033	0.76 (0.56–1.02), *p =* 0.07	0.84 (0.62–1.14), *p =* 0.27	0.87 (0.64–1.18), *p =* 0.376
*T*rend					0.019	0.044	0.199	0.297
Per SD increment	101,680	443	1,353,326.28	3.27	0.89 (0.79–0.99), *p =* 0.034	0.9 (0.81–1.01), *p =* 0.081	0.93 (0.83–1.03), *p =* 0.173	0.94 (0.84–1.05), *p =* 0.248
**Dietary** **α-carotene intakes, mcg/day**								
Q1 (<317.9)	20,336	102	265,741.25	3.84	1 (Ref)	1 (Ref)	1 (Ref)	1 (Ref)
Q2 (317.9–475.86)	20,336	91	268,878.31	3.38	0.88 (0.66–1.17), *p =* 0.375	0.88 (0.66–1.18), *p =* 0.402	0.95 (0.71–1.29), *p =* 0.755	0.95 (0.7–1.28), *p* = 0.742
Q3 (475.86–697.34)	20,336	90	269,982.27	3.33	0.87 (0.65–1.15), *p =* 0.319	0.87 (0.65–1.17), *p =* 0.358	0.96 (0.71–1.31), *p =* 0.811	0.96 (0.7–1.3), *p* = 0.772
Q4 (697.35–1135.29)	20,336	78	272,597.94	2.86	0.74 (0.55–1), *p =* 0.046	0.74 (0.55–1), *p =* 0.053	0.85 (0.6–1.19), *p =* 0.347	0.83 (0.6–1.16), *p* = 0.277
Q5 (>1135.37)	20,336	82	276,126.51	2.97	0.77 (0.57–1.03), *p =* 0.073	0.79 (0.58–1.06), *p =* 0.115	0.98 (0.6–1.61), *p =* 0.947	0.91 (0.62–1.35), *p* = 0.649
*T*rend					0.035	0.057	0.531	0.417
Per SD increment	101,680	443	1,353,326.28	3.27	0.9 (0.81–1), *p =* 0.06	0.92 (0.82–1.02), *p =* 0.113	0.94 (0.8–1.11), *p =* 0.475	0.94 (0.8–1.11), *p* = 0.49
**Dietary** **β-carotene intakes, mcg/day**								
Q1 (<1707.28)	20,336	103	264,087.73	3.90	1 (Ref)	1 (Ref)	1 (Ref)	1 (Ref)
Q2 (1707.28–2389.73)	20,336	101	267,252.54	3.778	0.97 (0.73–1.27), *p =* 0.806	0.98 (0.74–1.29), *p =* 0.866	1.05 (0.78–1.4), *p =* 0.759	1.06 (0.79–1.42), *p =* 0.705
Q3 (2389.74–3239.34)	20,336	76	271,162.62	2.80	0.71 (0.53–0.96), *p =* 0.026	0.74 (0.54–1), *p =* 0.048	0.81 (0.59–1.11), *p =* 0.184	0.82 (0.6–1.13), *p =* 0.225
Q4 (3239.4–4920.97)	20,336	82	273,732.67	3.00	0.76 (0.57–1.02), *p =* 0.064	0.79 (0.58–1.06), *p =* 0.116	0.88 (0.63–1.21), *p =* 0.422	0.9 (0.65–1.25), *p =* 0.521
Q5 (>4921.39)	20,336	81	277,090.73	2.92	0.74 (0.55–0.99), *p =* 0.042	0.77 (0.57–1.04), *p =* 0.093	0.88 (0.58–1.31), *p =* 0.52	0.91 (0.61–1.36), *p =* 0.64
*T*rend					0.01	0.033	0.253	0.344
Per SD increment	101,680	443	1,353,326.28	3.27	0.92 (0.83–1.02), *p =* 0.096	0.93 (0.84–1.04), *p =* 0.192	0.99 (0.85–1.16), *p =* 0.915	1.00 (0.86–1.17), *p =* 0.959

*PLCO, prostate, lung, colorectal, and ovarian; HR, hazard ratio; CI, confidence interval; SD, standard deviation*.

*Unadjusted was the crude model*.

*Model 1 adjusted for age (continuous) and sex (male vs. female)*.

*Model 2 adjusted for model 1 plus trial arm (intervention vs. control), race (white, non-Hispanic vs. black, non-Hispanic vs. Hispanic vs. others), marital status (married vs. unmarried), education level (college below vs. college graduate vs. postgraduate), aspirin use (yes vs. no), diabetes (yes vs. no), cigarette smoking (never vs. current vs. former), BMI (<2 vs. ≥25 kg/m^2^), family history of colorectal cancer (yes vs. no vs. possibly), energy intake from diet (continuous), alcohol drinking (never vs. former vs. current), supplemental Beta-Carotene (continuous)*.

*Model 3 adjusted for model 2 plus supplemental vitamin A (continuous), supplemental vitamin E (continuous), supplemental vitamin C (continuous), supplemental calcium (continuous)*.

*For the association of dietary α-carotene intake with colorectal cancer mortality, model 2 was further adjusted for energy-adjusted dietary β-carotene intake (mcg/day). For the association of dietary β-carotene intake with colorectal cancer mortality, model 2 was further adjusted for energy-adjusted dietary α-carotene intake (mcg/day)*.

## Discussion

In this prospective cohort of 101,680 U.S. adults, we found that only the quintile 4 group of dietary carrot intakes but not dietary α-, and β-carotene intakes, had a lower risk of colorectal cancer, compared with the referent group. Meanwhile, the null associations were also detected between dietary carrot, α-, and β-carotene intakes and colorectal cancer mortality. Similar results were supported in several sensitivity analyses. Of note, the association of dietary carrot intakes was modified by smoking status with colorectal cancer incidence but not mortality.

Although the effect of carrot consumption on multiple types of cancer risk has been widely investigated, the findings were mixed. In our analysis, except for the quintile 4 group of carrot intakes, however, there were no significant associations shown in other quintile groups. The observed inverse association of our study was close to a previous prospective cohort from Danes (17), in which they illustrated that a higher intake of raw carrots (>32 g/day) was related to a 17% lower risk of CRC, compared with no intake of raw carrots (HR = 0.83, 95%CI = 0.71–0.98), but as for the raw carrot intakes <32 g/day, no significant association was observed with decreased risk of CRC (HR = 0.93, 95%CI = 0.82–1.06). Of note, there were also some differences. Our results, based on a large prospective cohort of 101,680 US adults, found that moderate consumption of carrots (Q4, >7.60–15.30 g/day) but not the highest consumption of carrots (Q5, >15.30 g/day) was associated with a lower risk of CRC incidence, compared with the lowest consumption of carrot (Q1, <1.95 g/day), while the Danes cohort showed that a higher raw carrots intake was associated with a lower risk of CRC incidence. We further categorized the dietary carrot intake into three groups (0, 0–32 g/day, and ≥32 g/day), and the highest carrot intake was also null associated with CRC incidence (HR ≥32 g/day vs. none = 0.75, 95%CI = 0.46–1.24) after fully-adjustment. The small size of events might reduce the statistical power, in which only 56 (0.8%) incident cases and 26 (0.4%) CRC deaths were in the group of carrot intake ≥32 g/day, and lower the association with CRC risk. Some residual confounders, such as dietary habits, and assessment of exposure may account for the difference between the dose-response analyses of carrot consumption and CRC incidence in a different population and thus needed to interpret cautiously. In addition, we explored the association of carrot intake with CRC mortality, but no significant correlation was detected in the multivariable Cox models and other analyses.

The cancer-prevention effect of carrot intake might be explained that carrots are rich in carotenoid antioxidants such as α- and β-carotene, which showed a potential prevention effect on cancer development. Hence, we further examined the association between dietary α-, and β-carotene intakes and CRC incidence, and mortality, but no statistically significant inverse associations were observed, respectively. That was in line with the results of a meta-analysis of nine randomized controlled trials (32), in which no significant association was found between dietary intakes of α- and β-carotene and colorectal cancer incidence. However, another two case-control studies in the Chinese population (18, 19) noted an inverse association of dietary α- and β-carotene intake with the risk of colorectal cancer. The differences in retrospective study design, number of samples, exposure assessment, potential recall bias, and the unadjusted confounders may contribute to the conflict findings. Another biological interpretation might be that carrots are also a major source of falcarinol (FaOH), falcarindiol (FaDOH), which have been demonstrated to inhibit neoplastic transformations in the rat models with a prevention effect on the development of colorectal cancer (33, 34). Thus, our findings supported that the potential cancer-prevention effects on CRC might derive from other specific components, rather than α-, and β-carotene.

Interestingly, we found that smoking status was an effect modifier on the relationship between carrot intake and colorectal cancer incidence (*p* for interaction = 0.027). In the strata of never smokers, the HR of the risk of CRC was 0.99 (95%CI = 0.99–1, *p* < 0.01), even though the association was weak. The result was constant with the previous research that smoking as a well-established risk factor could attenuate or reversed the observed protective effect of dietary carotenoids on CRC occurrence (17).

There have several inevitable limitations in the current study. First, the nature of observational studies resulted in residual confounding that could not be fully ruled out. Second, the generalizability of conclusions may limit by the geographical location and population differences. Third, we could not take into account the dynamic change data because of the absence of repeated measurements of dietary nutrients. Forth, we lack the data on raw, cooked carrots, and carrot juice due to the limited raw data, thus we were impossible to further distinguish and analyze their effect on colorectal cancer risk. In addition, some epidemiological studies published inconsistent results about the association of serum α-, and β-carotene concentration with CRC incidence (18, 35). We were interested in this, but for the limited raw data, thus we were unable to further analyze the association between serum carrot/carotene and the risk of CRC.

In summary, this U.S. prospective cohort indicated that moderate consumption of dietary carrots was associated with a lower risk of CRC incidence, while no statistically significant associations were observed between dietary α-, and β-carotene intakes and CRC incidence. Dietary carrot, α-, and β-carotene intakes were null associated with CRC mortality. Furthermore, we found smoking status modified the association of dietary carrot intakes with CRC incidence but not mortality. The results added evidence for the potential cancer-prevention effect of dietary carrot intakes on CRC incidence but should be cautiously interpreted. More large, prospective, well-designed cohort studies are warranted to verify the findings in other populations.

## Data Availability Statement

The raw data supporting the conclusions of this article will be made available by the authors, without undue reservation.

## Ethics Statement

The PLCO study was approved by the Institutional Review Boards of the US National Cancer Institute and each study center and written informed consents were obtained from all eligible participants. The patients/participants provided their written informed consent to participate in this study.

## Author Contributions

ZJ: conception, design, and acquisition of data. WW, CF, and FL: administrative support. ZJ and HC: data analysis and interpretation. ZJ, CF, and ML: manuscript revising. All authors: manuscript writing, final approval, and accountable for all aspects of work ensuring integrity and accuracy.

## Conflict of Interest

The authors declare that the research was conducted in the absence of any commercial or financial relationships that could be construed as a potential conflict of interest.

## Publisher's Note

All claims expressed in this article are solely those of the authors and do not necessarily represent those of their affiliated organizations, or those of the publisher, the editors and the reviewers. Any product that may be evaluated in this article, or claim that may be made by its manufacturer, is not guaranteed or endorsed by the publisher.
