# Supplementary material for: Association of Dietary Carrot/Carotene Intakes With Colorectal Cancer Incidence and Mortality in the Prostate, Lung, Colorectal, and Ovarian Cancer Screening Trial
Source: Front Nutr. 2022 Jun 17;9:888898. doi: 10.3389/fnut.2022.888898 (PMC9247642; doi:10.3389/fnut.2022.888898)
Supplement: Supplementary file 1 [file Data_Sheet_1.docx]

| **Supplemental Table 1**. Subgroup analyses of the associations between energy-adjusted dietary carrot intakes and colorectal cancer incidence using multivariable cox regression analyses. | | | | | | |  |
| --- | --- | --- | --- | --- | --- | --- | --- |
|  |  |  |  |  |  |  |  |
| Subgroup | Total (n) | Cases, (n, %) | Adjusted HR (95%CI) | *P*-value | *P* for interaction |  |  |
| Age, years |  |  |  |  |  |  |  |
| <=60 | 24,465 | 159 (0.6) | 0.99 (0.98~1) | 0.13 | 0.135 |  |  |
| >60 | 77,215 | 941 (1.2) | 1 (0.99~1) | 0.331 |  |  |  |
| Sex |  |  |  |  |  |  |  |
| Male | 49,441 | 608 (1.2) | 1 (0.99~1.01) | 0.999 | 0.1 |  |  |
| Female | 52,239 | 492 (0.9) | 0.99 (0.99~1) | 0.036 |  |  |  |
| Race |  |  |  |  |  |  |  |
| White, Non-Hispanic | 92,465 | 990 (1.1) | 1 (0.99~1) | 0.148 | 0.627 |  |  |
| Black, Non-Hispanic | 3,352 | 48 (1.4) | 1.01 (0.99~1.03) | 0.392 |  |  |  |
| Hispanic | 1,493 | 13 (0.9) | 0.98 (0.92~1.04) | 0.525 |  |  |  |
| Others | 4,333 | 49 (1.1) | 1 (0.97~1.02) | 0.761 |  |  |  |
| Marital status |  |  |  |  |  |  |  |
| Married | 79,578 | 851 (1.1) | 1 (0.99~1) | 0.42 | 0.224 |  |  |
| Unmarried | 21,916 | 245 (1.1) | 0.99 (0.98~1) | 0.093 |  |  |  |
| Trial arm |  |  |  |  |  |  |  |
| Intervention | 51,767 | 444 (0.9) | 1 (0.99~1) | 0.725 | 0.208 |  |  |
| Control | 49,913 | 656 (1.3) | 1 (0.99~1) | 0.087 |  |  |  |
| Education level |  |  |  |  |  |  |  |
| College below | 64,704 | 746 (1.2) | 1 (0.99~1) | 0.305 | 0.771 |  |  |
| College graduate | 17,838 | 193 (1.1) | 0.99 (0.99~1) | 0.257 |  |  |  |
| Postgraduate | 18,941 | 158 (0.8) | 1 (0.99~1.01) | 0.638 |  |  |  |
| Aspirin use |  |  |  |  |  |  |  |
| No | 53,472 | 599 (1.1) | 0.99 (0.99~1) | 0.042 | 0.2 |  |  |
| Yes | 47,775 | 495 (1) | 1 (0.99~1.01) | 0.981 |  |  |  |
| Diabetes |  |  |  |  |  |  |  |
| No | 94,353 | 991 (1.1) | 1 (0.99~1) | 0.15 | 0.789 |  |  |
| Yes | 6,801 | 106 (1.6) | 1 (0.98~1.01) | 0.689 |  |  |  |
| Cigarette smoking |  |  |  |  |  |  |  |
| Never | 48,532 | 485 (1) | 0.99 (0.99~1) | 0.01 | **0.027** |  |  |
| Current | 9,393 | 108 (1.1) | 1.01 (1~1.02) | 0.197 |  |  |  |
| Former | 43,742 | 507 (1.2) | 1 (0.99~1.01) | 0.954 |  |  |  |
| BMI, kg/m^2^ |  |  |  |  |  |  |  |
| <25 | 34,426 | 334 (1) | 0.99 (0.99~1) | 0.119 | 0.316 |  |  |
| >=25 | 65,915 | 743 (1.1) | 1 (0.99~1) | 0.443 |  |  |  |
| Family history of colorectal cancer | |  |  |  |  |  |  |
| No | 88,113 | 928 (1.1) | 1 (0.99~1) | 0.164 | 0.804 |  |  |
| Yes | 10,300 | 123 (1.2) | 1 (0.98~1.01) | 0.463 |  |  |  |
| Possibly | 2,493 | 40 (1.6) | 0.99 (0.97~1.02) | 0.574 |  |  |  |
| Alcohol drinking |  |  |  |  |  |  |  |
| Never | 10,110 | 97 (1) | 1 (0.98~1.01) | 0.463 | 0.488 |  |  |
| Former | 14,746 | 165 (1.1) | 1 (0.99~1.01) | 0.699 |  |  |  |
| Current | 73,944 | 809 (1.1) | 1 (0.99~1) | 0.074 |  |  |  |
| HR, hazard ratio; CI, confidence interval; BMI, body mass index. | | | | | | |  |
| Each subgroup adjusted for age (<=60 vs. >60), sex (male vs. female), trial arm (intervention vs. control), race (white, non-Hispanic vs. black, non-Hispanic vs. Hispanic vs. others), marital status (married vs. unmarried), education level (college below vs. college graduate vs. postgraduate), aspirin use (yes vs. no), diabetes (yes vs. no), cigarette smoking (never vs. current vs. former), BMI (<25kg/m^2^ vs. >=25kg/m^2^)，family history of colorectal cancer (yes vs. no vs. possibly), energy intake from diet (continuous), alcohol drinking (never vs. former vs. current),supplemental Beta-Carotene (continuous), supplemental vitamin A (continuous), supplemental vitamin E (continuous), supplemental vitamin C (continuous), and supplemental calcium (continuous), except the stratification factor itself. P for interaction was calculated using a likelihood ratio test. | | | | | | |  |
|  |  |  |  |  |  |  |  |
|  |  |  |  |  |  |  |  |
|  |  |  |  |  |  |  |  |
|  |  |  |  |  |  |  |  |
|  |  |  |  |  |  |  |  |
|  |  |  |  |  |  |  |  |
|  |  |  |  |  |  |  |  |

| **Supplemental Table 2**. Subgroup analyses of the associations between dietary carrot intakes and colorectal cancer mortality. | | | | | | | | | | | | |  |  |  |
| --- | --- | --- | --- | --- | --- | --- | --- | --- | --- | --- | --- | --- | --- | --- | --- |
|  |  |  |  |  |  |  |  |  |  |  |  |  |  |  |  |
| Subgroup | | | Total (n) | Cases, (n, %) | | | Adjusted HR (95%CI) | | *P*-value | *P* for interaction | | |  |  |  |
| Age, years | | |  |  | | |  | |  |  | | |  |  |  |
| <=60 | | | 24,465 | 59 (0.2) | | | 0.99 (0.97~1.01) | | 0.347 | 0.551 | | |  |  |  |
| >60 | | | 77,215 | 384 (0.5) | | | 1 (0.99~1) | | 0.439 |  | | |  |  |  |
| Sex | | |  |  | | |  | |  |  | | |  |  |  |
| Male | | | 49,441 | 255 (0.5) | | | 0.99 (0.99~1) | | 0.276 | 0.561 | | |  |  |  |
| Female | | | 52,239 | 188 (0.4) | | | 1 (0.99~1.01) | | 0.812 |  | | |  |  |  |
| Race | | |  |  | | |  | |  |  | | |  |  |  |
| White, Non-Hispanic | | | 92,465 | 387 (0.4) | | | 1 (0.99~1) | | 0.368 | 0.708 | | |  |  |  |
| Black, Non-Hispanic | | | 3,352 | 31 (0.9) | | | 1.01 (0.98~1.03) | | 0.511 |  | | |  |  |  |
| Hispanic | | | 1,493 | 9 (0.6) | | | 0.99 (0.92~1.05) | | 0.704 |  | | |  |  |  |
| Others | | | 4,333 | 16 (0.4) | | | 0.99 (0.94~1.04) | | 0.753 |  | | |  |  |  |
| Marital status | | |  |  | | |  | |  |  | | |  |  |  |
| Married | | | 79,578 | 321 (0.4) | | | 1 (0.99~1.01) | | 0.606 | 0.431 | | |  |  |  |
| Unmarried | | | 21,916 | 119 (0.5) | | | 0.99 (0.98~1.01) | | 0.296 |  | | |  |  |  |
| Trial arm | | |  |  | | |  | |  |  | | |  |  |  |
| Intervention | | | 51,767 | 174 (0.3) | | | 0.99 (0.98~1) | | 0.152 | 0.347 | | |  |  |  |
| Control | | | 49,913 | 269 (0.5) | | | 1 (0.99~1.01) | | 0.839 |  | | |  |  |  |
| Education level | | |  |  | | |  | |  |  | | |  |  |  |
| College below | | | 64,704 | 286 (0.4) | | | 1 (0.99~1) | | 0.447 | 0.654 | | |  |  |  |
| College graduate | | | 17,838 | 79 (0.4) | | | 1 (0.99~1.01) | | 0.941 |  | | |  |  |  |
| Postgraduate | | | 18,941 | 75 (0.4) | | | 0.99 (0.98~1.01) | | 0.295 |  | | |  |  |  |
| Aspirin use | | |  |  | | |  | |  |  | | |  |  |  |
| No | | | 53,472 | 237 (0.4) | | | 0.99 (0.99~1) | | 0.248 | 0.7 | | |  |  |  |
| Yes | | | 47,775 | 204 (0.4) | | | 1 (0.99~1.01) | | 0.777 |  | | |  |  |  |
| Diabetes | | |  |  | | |  | |  |  | | |  |  |  |
| No | | | 94,353 | 400 (0.4) | | | 1 (0.99~1) | | 0.374 | 0.739 | | |  |  |  |
| Yes | | | 6,801 | 41 (0.6) | | | 0.99 (0.97~1.02) | | 0.588 |  | | |  |  |  |
| Cigarette smoking | | |  |  | | |  | |  |  | | |  |  |  |
| Never | | | 48,532 | 183 (0.4) | | | 0.99 (0.98~1) | | 0.054 | 0.19 | | |  |  |  |
| Current | | | 9,393 | 50 (0.5) | | | 1 (0.98~1.02) | | 0.988 |  | | |  |  |  |
| Former | | | 43,742 | 210 (0.5) | | | 1 (0.99~1.01) | | 0.616 |  | | |  |  |  |
| BMI, kg/m^2^ | | |  |  | | |  | |  |  | | |  |  |  |
| <25 | | | 34,426 | 129 (0.4) | | | 1 (0.99~1.01) | | 0.941 | 0.63 | | |  |  |  |
| >=25 | | | 65,915 | 304 (0.5) | | | 1 (0.99~1) | | 0.312 |  | | |  |  |  |
| Family history of colorectal cancer | | | |  | | |  | |  |  | | |  |  |  |
| No | | | 88,113 | 371 (0.4) | | | 1 (0.99~1) | | 0.334 | 0.712 | | |  |  |  |
| Yes | | | 10,300 | 47 (0.5) | | | 1 (0.98~1.02) | | 0.766 |  | | |  |  |  |
| Possibly | | | 2,493 | 22 (0.9) | | | 0.98 (0.94~1.03) | | 0.469 |  | | |  |  |  |
| Alcohol drinking | | |  |  | | |  | |  |  | | |  |  |  |
| Never | | | 10,110 | 38 (0.4) | | | 0.99 (0.97~1.02) | | 0.658 | 0.297 | | |  |  |  |
| Former | | | 14,746 | 67 (0.5) | | | 1 (0.99~1.02) | | 0.477 |  | | |  |  |  |
| Current | | | 73,944 | 329 (0.4) | | | 1 (0.99~1) | | 0.209 |  | | |  |  |  |
| HR, hazard ratio; CI, confidence interval; BMI, body mass index.  Each subgroup adjusted for age (<=60 vs. >60), sex (male vs. female), trial arm (intervention vs. control), race (white, non-Hispanic vs. black, non-Hispanic vs. Hispanic vs. others), marital status (married vs. unmarried), education level (college below vs. college graduate vs. postgraduate), aspirin use (yes vs. no), diabetes (yes vs. no), cigarette smoking (never vs. current vs. former), BMI (<25kg/m^2^ vs. >=25kg/m^2^)，family history of colorectal cancer (yes vs. no vs. possibly), energy intake from diet (continuous), alcohol drinking (never vs. former vs. current),supplemental Beta-Carotene (continuous), supplemental vitamin A (continuous), supplemental vitamin E (continuous), supplemental vitamin C (continuous), and supplemental calcium (continuous), except the stratification factor itself. *P* for interaction was calculated using a likelihood ratio test. | | | | | | | | | | | | |  |  |  |
|  |  |  |  |  |  |  |  |  |  |  |  |  |  |  |  |
|  | | |  |  | | |  | |  |  | | |  |  |  |
| **Supplemental Table 3**. Sensitivity analyses on the association between dietary carrot/carotene intakes and colorectal cancer incidence | | | | | | | | | | | | | |  |  |
| Quintile of dietary carrot/carotene intakes | Hazard ratio (95% confidence interval) ^a^ | | | | | | | | | | | | |  |  |
|  | Primary analysis | | | | Sensitivity analyses | | | | | | | | |  |  |
|  |  |  |  |  | Excluding cases occurring within the first five years of follow-up | | | Excluding subjects with extreme values of energy intake | | | additional adjusted for other factors ^b^ | | |  |  |
| Dietary carrot intakes, g/day | | | | |  | | |  | | |  | | |  |  |
| Quintile 1 | 1(Ref) | | | | 1(Ref) | | | 1(Ref) | | | 1(Ref) | | |  |  |
| Quintile 2 | 1 (0.82~1.21), p=0.995 | | | | 1.27 (0.93~1.73), p=0.134 | | | 1.01 (0.83~1.23), p=0.956 | | | 1.01 (0.83~1.23), p=0.933 | | |  |  |
| Quintile 3 | 1 (0.82~1.21), p=0.968 | | | | 1.27 (0.93~1.73), p=0.132 | | | 1.01 (0.83~1.23), p=0.931 | | | 1 (0.82~1.23), p=0.965 | | |  |  |
| Quintile 4 | 0.79 (0.65~0.97), p=0.025 | | | | 1.14 (0.84~1.56), p=0.405 | | | 0.81 (0.66~0.99), p=0.043 | | | 0.81 (0.66~1), p=0.047 | | |  |  |
| Quintile 5 | 0.9 (0.74~1.1), p=0.306 | | | | 1.12 (0.82~1.55), p=0.468 | | | 0.92 (0.75~1.13), p=0.431 | | | 0.93 (0.74~1.15), p=0.487 | | |  |  |
| *T*_rend_ | 0.05 | | | | 0.814 | | | 0.094 | | | 0.118 | | |  |  |
| Per SD increment | 0.95 (0.89~1.02), p=0.169 | | | | 1.01 (0.91~1.11)，p=0.893 | | | 0.96 (0.9~1.03), p=0.245 | | | 0.97 (0.89~1.04)， p=0.357 | | |  |  |
|  |  | | | |  | | |  | | |  | | |  |  |
| Dietary α-carotene intakes, mcg/day | | | | |  | | |  | | |  | | |  |  |
| Quintile 1 | 1(Ref) | | | | 1(Ref) | | | 1(Ref) | | | 1(Ref) | | |  |  |
| Quintile 2 | 1.02 (0.84~1.24), p=0.82 | | | | 1.35 (0.99~1.83), p=0.056 | | | 1.05 (0.86~1.27), p=0.651 | | | 1.02 (0.84~1.23), p=0.872 | | |  |  |
| Quintile 3 | 1.01 (0.83~1.23), p=0.892 | | | | 1.32 (0.97~1.8), p=0.082 | | | 1.02 (0.84~1.25), p=0.815 | | | 1 (0.82~1.23), p=0.97 | | |  |  |
| Quintile 4 | 0.83 (0.67~1.03), p=0.087 | | | | 1.07 (0.76~1.49), p=0.706 | | | 0.84 (0.67~1.04), p =0.111 | | | 0.82 (0.66~1.02), p=0.081 | | |  |  |
| Quintile 5 | 0.97 (0.76~1.25), p=0.832 | | | | 1.09 (0.73~1.62), p=0.676 | | | 1.04 (0.8~1.35), p=0.756 | | | 0.97 (0.75~1.26), p=0.819 | | |  |  |
| *T*_rend_ | 0.249 | | | | 0.878 | | | 0.419 | | | 0.245 | | |  |  |
| Per SD increment | 0.99 (0.89~1.1), p=0.833 | | | | 1.01 (0.87~1.18), p=0.889 | | | 1.02 (0.91~1.14), p=0.75 | | | 1 (0.89~1.11), p=0.95 | | |  |  |
|  |  | | | |  | | |  | | |  | | |  |  |
| Dietary β-carotene intakes, mcg/day | | | | |  | | |  | | |  | | |  |  |
| Quintile 1 | 1(Ref) | | | | 1(Ref) | | | 1(Ref) | | | 1(Ref) | | |  |  |
| Quintile 2 | 1.07 (0.89~1.29), p=0.477 | | | | 1.27 (0.95~1.69), p=0.113 | | | 1.09 (0.91~1.32), p=0.351 | | | 1.06 (0.87~1.28), p=0.563 | | |  |  |
| Quintile 3 | 0.87 (0.71~1.07), p=0.177 | | | | 0.92 (0.67~1.27), p=0.605 | | | 0.88 (0.72~1.09), p=0.243 | | | 0.85 (0.69~1.06), p=0.148 | | |  |  |
| Quintile 4 | 1.01 (0.83~1.24), p=0.893 | | | | 1.15 (0.84~1.58), p=0.383 | | | 1 (0.81~1.24), p=0.984 | | | 0.99 (0.79~1.24), p=0.929 | | |  |  |
| Quintile 5 | 0.84 (0.64~1.1), p=0.197 | | | | 0.87 (0.58~1.32), p=0.519 | | | 0.86 (0.65~1.13), p=0.269 | | | 0.8 (0.59~1.09), p=0.16 | | |  |  |
| *T*_rend_ | 0.243 | | | | 0.561 | | | 0.241 | | | 0.208 | | |  |  |
| Per SD increment | 0.97 (0.87~1.07), p=0.519 | | | | 1 (0.86~1.18), p=0.957 | | | 0.94 (0.84~1.05), p=0.257 | | | 0.96 (0.83~1.1), p=0.569 | | |  |  |
| SD, standard deviation.  ^a^ Adjusted for age (continuous) and sex (male vs. female), trial arm (intervention vs. control), race (white, non-Hispanic vs. black, non-Hispanic vs. Hispanic vs. others), marital status (married vs. unmarried), education level (college below vs. college graduate vs. postgraduate), aspirin use (yes vs. no), diabetes (yes vs. no), cigarette smoking (never vs. current vs. former), BMI (<25kg/m^2^ vs. >=25kg/m^2^)，family history of colorectal cancer (yes vs. no vs. possibly), energy intake from diet (continuous), alcohol drinking (never vs. former vs. current), supplemental Beta-Carotene (continuous).supplemental vitamin A (continuous), supplemental vitamin E (continuous), supplemental vitamin C (continuous), and supplemental calcium (continuous). For the association of dietary α-carotene intake with colorectal cancer incidence, we further adjusted for energy-adjusted dietary β-carotene intake (mcg/day). For the association of dietary β-carotene intake with colorectal cancer incidence, we further adjusted for energy-adjusted dietary α-carotene intake (mcg/day). ^b^ included fruit (continuous), dietary Magnesium (continuous), dietary Sodium (continuous), dietary Potassium (continuous), whole grain (continuous), vegetables (continuous), added sugars (continuous), fiber (continuous), and saturated fatty acids (continuous). | | | | | | | | | | | | | |  |  |
|  |  |  |  |  |  |  |  |  |  |  |  |  |  |  | |
|  |  |  |  |  |  |  |  |  |  |  |  |  |  |  | |
|  |  |  |  |  |  |  |  |  |  |  |  |  |  |  | |
|  |  |  |  |  |  |  |  |  |  |  |  |  |  |  | |
|  |  |  |  |  |  |  |  |  |  |  |  |  |  |  | |
|  |  |  |  |  |  |  |  |  |  |  |  |  |  |  | |
|  |  |  |  |  |  |  |  |  |  |  |  |  |  |  | |
|  |  |  |  |  |  |  |  |  |  |  |  |  |  |  | |
|  |  |  |  |  |  |  |  |  |  |  |  |  |  |  | |
|  |  |  |  |  |  |  |  |  |  |  |  |  |  |  | |
|  |  | | | |  | | |  | | |  | | |  | |
| **Supplemental Table 4**. Sensitivity analyses on the association between dietary carrot/carotene intakes and colorectal cancer mortality | | | | | | | | | | | | | | |  |
| Quintile of dietary carrot/carotene intakes | | Hazard ratio (95% confidence interval) ^a^ | | | | | | | | | | | | |  |
|  |  | Primary analysis | | | | Sensitivity analyses | | | | | | | | |  |
|  |  |  |  |  |  | Excluding cases occurring within the first five years of follow-up | | Excluding subjects with extreme values of energy intake | | | | additional adjusted for other factors ^b^ | | |  |
| Dietary carrot intakes, g/day | | | | | |  | |  | | | |  | | |  |
| Quintile 1 | | 1(Ref) | | | | 1(Ref) | | 1(Ref) | | | | 1(Ref) | | |  |
| Quintile 2 | | 0.94 (0.7~1.28), p=0.71 | | | | 0.94 (0.66~1.33), p=0.714 | | 0.9 (0.65~1.23), p=0.502 | | | | 0.96 (0.71~1.31), p=0.814 | | |  |
| Quintile 3 | | 0.91 (0.67~1.24), p=0.569 | | | | 0.88 (0.61~1.26), p=0.475 | | 0.91 (0.66~1.25), p=0.551 | | | | 0.93 (0.68~1.27), p=0.652 | | |  |
| Quintile 4 | | 0.87 (0.64~1.18), p=0.359 | | | | 0.97 (0.69~1.37), p=0.872 | | 0.91 (0.67~1.25), p=0.567 | | | | 0.9 (0.66~1.25), p=0.54 | | |  |
| Quintile 5 | | 0.87 (0.64~1.18), p=0.376 | | | | 1.04 (0.74~1.47), p=0.803 | | 0.87 (0.63~1.21), p=0.412 | | | | 0.92 (0.65~1.3), p=0.635 | | |  |
| *T*_rend_ | | 0.297 | | | | 0.761 | | 0.499 | | | | 0.549 | | |  |
| Per SD increment | | 0.94 (0.84~1.05), p=0.248 | | | | 1 (0.9~1.12), p=0.967 | | 0.95 (0.85~1.06), p=0.367 | | | | 0.96 (0.85~1.08), p=0.45 | | |  |
|  | |  | | | |  | |  | | | |  | | |  |
| Dietary α-carotene intakes, mcg/day | | | | | |  | |  | | | |  | | |  |
| Quintile 1 | | 1(Ref) | | | | 1(Ref) | | 1(Ref) | | | | 1(Ref) | | |  |
| Quintile 2 | | 0.95 (0.7~1.28), p=0.742 | | | | 0.97 (0.68~1.37), p=0.847 | | 0.99 (0.73~1.35), p=0.94 | | | | 0.94 (0.69~1.27), p=0.688 | | |  |
| Quintile 3 | | 0.96 (0.7~1.3), p=0.772 | | | | 0.92 (0.64~1.32), p=0.646 | | 0.95 (0.69~1.31), p=0.744 | | | | 0.94 (0.68~1.29), p=0.69 | | |  |
| Quintile 4 | | 0.83 (0.6~1.16), p=0.277 | | | | 0.96 (0.66~1.38), p=0.818 | | 0.93 (0.66~1.31), p=0.67 | | | | 0.82 (0.58~1.15), p=0.248 | | |  |
| Quintile 5 | | 0.91 (0.62~1.35), p=0.649 | | | | 1.07 (0.69~1.66), p=0.755 | | 1.1 (0.73~1.68), p=0.644 | | | | 0.9 (0.6~1.35), p=0.614 | | |  |
| *T*_rend_ | | 0.417 | | | | 0.908 | | 0.96 | | | | 0.39 | | |  |
| Per SD increment | | 0.94 (0.8~1.11), p=0.49 | | | | 1.03 (0.86~1.23), p=0.746 | | 1.01 (0.84~1.2), p=0.956 | | | | 0.95 (0.8~1.12), p=0.533 | | |  |
|  | |  | | | |  | |  | | | |  | | |  |
| Dietary β-carotene intakes, mcg/day | | | | | |  | |  | | | |  | | |  |
| Quintile 1 | | 1(Ref) | | | | 1(Ref) | | 1(Ref) | | | | 1(Ref) | | |  |
| Quintile 2 | | 1.06 (0.79~1.42), p=0.705 | | | | 0.92 (0.65~1.28), p=0.605 | | 1.06 (0.79~1.43), p=0.689 | | | | 1.03 (0.76~1.39), p=0.854 | | |  |
| Quintile 3 | | 0.82 (0.6~1.13), p=0.225 | | | | 0.71 (0.49~1.03), p=0.072 | | 0.8 (0.57~1.11), p=0.181 | | | | 0.79 (0.56~1.1), p=0.16 | | |  |
| Quintile 4 | | 0.9 (0.65~1.25), p=0.521 | | | | 0.89 (0.62~1.27), p=0.513 | | 0.92 (0.65~1.28), p=0.611 | | | | 0.85 (0.6~1.22), p=0.384 | | |  |
| Quintile 5 | | 0.91 (0.61~1.36), p=0.64 | | | | 0.84 (0.54~1.31), p=0.443 | | 0.94 (0.62~1.43), p=0.773 | | | | 0.84 (0.53~1.34), p=0.464 | | |  |
| *T*_rend_ | | 0.344 | | | | 0.361 | | 0.429 | | | | 0.226 | | |  |
| Per SD increment | | 1.00 (0.86~1.17), p=0.959 | | | | 0.98 (0.82~1.17), p=0.785 | | 0.93 (0.78~1.12), p=0.442 | | | | 1.02 (0.83~1.25), p=0.871 | | |  |
| SD, standard deviation. | | | | | | | | | | | | | | |  |
| ^a^ Adjusted for age (continuous) and sex (male vs. female), trial arm (intervention vs. control), race (white, non-Hispanic vs. black, non-Hispanic vs. Hispanic vs. others), marital status (married vs. unmarried), education level (college below vs. college graduate vs. postgraduate), aspirin use (yes vs. no), diabetes (yes vs. no), cigarette smoking (never vs. current vs. former), BMI (<25kg/m^2^ vs. >=25kg/m^2^)，family history of colorectal cancer (yes vs. no vs. possibly), energy intake from diet (continuous), alcohol drinking (never vs. former vs. current),supplemental Beta-Carotene (continuous).supplemental vitamin A (continuous), supplemental vitamin E (continuous), supplemental vitamin C (continuous), and supplemental calcium (continuous). For the association of dietary α-carotene intake with colorectal cancer mortality, we further adjusted for energy-adjusted dietary β-carotene intake (mcg/day). For the association of dietary β-carotene intake with colorectal cancer mortality, we further adjusted for energy-adjusted dietary α-carotene intake (mcg/day). ^b^ included fruit (continuous), dietary Magnesium (continuous), dietary Sodium (continuous), dietary Potassium (continuous), whole grain (continuous), vegetables (continuous), added sugars (continuous), fiber (continuous), and saturated fatty acids (continuous). | | | | | | | | | | | | | | |  |
|  |  |  |  |  |  |  |  |  |  |  |  |  |  |  |  |
|  |  |  |  |  |  |  |  |  |  |  |  |  |  |  |  |
|  |  |  |  |  |  |  |  |  |  |  |  |  |  |  |  |
|  |  |  |  |  |  |  |  |  |  |  |  |  |  |  |  |
|  |  |  |  |  |  |  |  |  |  |  |  |  |  |  |  |
|  |  |  |  |  |  |  |  |  |  |  |  |  |  |  |  |
|  |  |  |  |  |  |  |  |  |  |  |  |  |  |  |  |
|  | |  | | | |  | |  | | | |  | | |  |

| **Supplement Table 5**. Baseline characteristics of non-steroidal anti-inflammatory drugs and some diseases according to the quintiles of carrot intakes. | | | | | | | |
| --- | --- | --- | --- | --- | --- | --- | --- |
|  |  | Quintiles of energy-adjusted dietary carrot intakes, g/day | | | | |  |
| Variables | Total (n = 101680) | Q1 (n = 20336) | Q2 (n = 20336) | Q3 (n = 20336) | Q4 (n = 20336) | Q5 (n = 20336) | *P* |
| Aspirin use, n (%) |  |  |  |  |  |  | < 0.001 |
| No | 53472 (52.6) | 10082 (49.6) | 10763 (52.9) | 10849 (53.3) | 10789 (53.1) | 10989 (54) |  |
| Yes | 47775 (47.0) | 10148 (49.9) | 9475 (46.6) | 9401 (46.2) | 9467 (46.6) | 9284 (45.7) |  |
| Missing | 433 (0.4) | 106 (0.5) | 98 (0.5) | 86 (0.4) | 80 (0.4) | 63 (0.3) |  |
| Ibuprofen use, n (%) |  |  |  |  |  |  | 0.027 |
| No | 72861 (71.7) | 14737 (72.5) | 14634 (72) | 14538 (71.5) | 14542 (71.5) | 14410 (70.9) |  |
| Yes | 28392 (27.9) | 5518 (27.1) | 5622 (27.6) | 5698 (28) | 5712 (28.1) | 5842 (28.7) |  |
| Missing | 427 (0.4) | 81 (0.4) | 80 (0.4) | 100 (0.5) | 82 (0.4) | 84 (0.4) |  |
| Diabetes, n (%) |  |  |  |  |  |  | < 0.001 |
| No | 94353 (92.8) | 18702 (92) | 18695 (91.9) | 18867 (92.8) | 19002 (93.4) | 19087 (93.9) |  |
| Yes | 6801 (6.7) | 1536 (7.6) | 1525 (7.5) | 1353 (6.7) | 1241 (6.1) | 1146 (5.6) |  |
| Missing | 526 (0.5) | 98 (0.5) | 116 (0.6) | 116 (0.6) | 93 (0.5) | 103 (0.5) |  |
| colon comorbidity, n (%) |  |  |  |  |  |  | 0.105 |
| No | 99439 (97.8) | 19873 (97.7) | 19884 (97.8) | 19858 (97.6) | 19913 (97.9) | 19911 (97.9) |  |
| Yes | 1355 (1.3) | 301 (1.5) | 262 (1.3) | 290 (1.4) | 261 (1.3) | 241 (1.2) |  |
| Missing | 886 (0.9) | 162 (0.8) | 190 (0.9) | 188 (0.9) | 162 (0.8) | 184 (0.9) |  |
| Gallbladder stones or inflammation, n (%) | |  |  |  |  |  | < 0.001 |
| No | 89489 (88.0) | 18334 (90.2) | 17824 (87.6) | 17760 (87.3) | 17753 (87.3) | 17818 (87.6) |  |
| Yes | 11564 (11.4) | 1888 (9.3) | 2373 (11.7) | 2439 (12) | 2469 (12.1) | 2395 (11.8) |  |
| Missing | 627 (0.6) | 114 (0.6) | 139 (0.7) | 137 (0.7) | 114 (0.6) | 123 (0.6) |  |
| Heart attack, n (%) |  |  |  |  |  |  | < 0.001 |
| No | 92716 (91.2) | 18254 (89.8) | 18368 (90.3) | 18600 (91.5) | 18703 (92) | 18791 (92.4) |  |
| Yes | 8430 (8.3) | 1988 (9.8) | 1852 (9.1) | 1618 (8) | 1538 (7.6) | 1434 (7.1) |  |
| Missing | 534 (0.5) | 94 (0.5) | 116 (0.6) | 118 (0.6) | 95 (0.5) | 111 (0.5) |  |
| Stroke, n (%) |  |  |  |  |  |  | 0.006 |
| No | 99109 (97.5) | 19793 (97.3) | 19783 (97.3) | 19790 (97.3) | 19882 (97.8) | 19861 (97.7) |  |
| Yes | 2044 (2.0) | 446 (2.2) | 432 (2.1) | 438 (2.2) | 360 (1.8) | 368 (1.8) |  |
| Missing | 527 (0.5) | 97 (0.5) | 121 (0.6) | 108 (0.5) | 94 (0.5) | 107 (0.5) |  |
| Colorectal polyps, n (%) |  |  |  |  |  |  | 0.007 |
| No | 94305 (92.7) | 18794 (92.4) | 18880 (92.8) | 18856 (92.7) | 18827 (92.6) | 18948 (93.2) |  |
| Yes | 6762 (6.7) | 1428 (7) | 1320 (6.5) | 1350 (6.6) | 1407 (6.9) | 1257 (6.2) |  |
| Missing | 613 (0.6) | 114 (0.6) | 136 (0.7) | 130 (0.6) | 102 (0.5) | 131 (0.6) |  |
|  |  |  |  |  |  |  |  |

| **Supplementary Table 6**. Additional analyses on the associations between energy-adjusted dietary carrot/carotene intake and colorectal cancer incidence risk in the PLCO cancer screening trial. | | | | | | | | | | | |
| --- | --- | --- | --- | --- | --- | --- | --- | --- | --- | --- | --- |
|  | Hazard ratios (95% confidence interval), *P*-value | | | | | | | | | | |
|  | **only adjusted aspirin** | |  | **not adjusted aspirin** | |  | **only adjusted ibuprofen** | |  | **adjusted aspirin and ibuprofen** | |
| Variables | **Model 3** | **Model 4** |  | **Model 5** | **Model 6** |  | **Model 7** | **Model 8** |  | **Model 9** | **Model 10** |
|  |  |  |  |  |  |  |  |  |  |  |  |
| Dietary carrot intake, g/day | |  |  |  |  |  |  |  |  |  |  |
| Q1 | 1(Ref) | 1(Ref) |  | 1(Ref) | 1(Ref) |  | 1(Ref) | 1(Ref) |  | 1(Ref) | 1(Ref) |
| Q2 | 1 (0.82~1.21), p=0.995 | 1 (0.83~1.22), p=0.982 |  | 1 (0.83~1.22), p=0.986 | 1 (0.83~1.22), p=0.987 |  | 1 (0.83~1.22), p=0.986 | 1 (0.83~1.22), p=0.987 |  | 1 (0.82~1.21), p=0.996 | 1 (0.82~1.21), p=0.996 |
| Q3 | 1 (0.82~1.21), p=0.968 | 1 (0.82~1.21), p=0.975 |  | 1 (0.82~1.21), p=0.981 | 1 (0.82~1.21), p=0.977 |  | 1 (0.82~1.21), p=0.981 | 1 (0.82~1.21), p=0.976 |  | 1 (0.82~1.21), p=0.975 | 1 (0.82~1.21), p=0.971 |
| Q4 | 0.79 (0.65~0.97), p=0.025 | 0.8 (0.65~0.97), p=0.027 |  | 0.79 (0.65~0.97), p=0.026 | 0.79 (0.65~0.97), p=0.025 |  | 0.79 (0.65~0.97), p=0.026 | 0.79 (0.65~0.97), p=0.025 |  | 0.79 (0.65~0.97), p=0.025 | 0.79 (0.65~0.97), p=0.025 |
| Q5 | 0.9 (0.74~1.1), p=0.306 | 0.9 (0.74~1.1), p=0.309 |  | 0.9 (0.74~1.1), p=0.315 | 0.9 (0.74~1.1), p=0.319 |  | 0.9 (0.74~1.1), p=0.315 | 0.9 (0.74~1.1), p=0.32 |  | 0.9 (0.74~1.1), p=0.31 | 0.9 (0.74~1.1), p=0.315 |
| *T*rend | 0.05 | 0.051 |  | 0.051 | 0.052 |  | 0.051 | 0.052 |  | 0.051 | 0.052 |
| Per SD increment | 0.95 (0.89~1.02), p=0.169 | 0.95 (0.89~1.02), p=0.169 |  | 0.95 (0.89~1.02), p=0.169 | 0.95 (0.89~1.02), p=0.174 |  | 0.95 (0.89~1.02), p=0.169 | 0.95 (0.89~1.02), p=0.174 |  | 0.95 (0.89~1.02), p=0.169 | 0.95 (0.89~1.02), p=0.174 |
|  |  |  |  |  |  |  |  |  |  |  |  |
| Dietary α-carotene intake, mcg/day | |  |  |  |  |  |  |  |  |  |  |
| Q1 | 1(Ref) | 1(Ref) |  | 1(Ref) | 1(Ref) |  | 1(Ref) | 1(Ref) |  | 1(Ref) | 1(Ref) |
| Q2 | 1.02 (0.84~1.24), p=0.82 | 1.02 (0.84~1.24), p=0.811 |  | 1.02 (0.84~1.24), p=0.825 | 1.02 (0.84~1.24), p=0.826 |  | 1.02 (0.84~1.24), p=0.825 | 1.02 (0.84~1.24), p=0.826 |  | 1.02 (0.84~1.24), p=0.818 | 1.02 (0.84~1.24), p=0.819 |
| Q3 | 1.01 (0.83~1.23), p=0.892 | 1.01 (0.83~1.24), p=0.883 |  | 1.01 (0.83~1.23), p=0.902 | 1.01 (0.83~1.23), p=0.902 |  | 1.01 (0.83~1.23), p=0.902 | 1.01 (0.83~1.23), p=0.902 |  | 1.01 (0.83~1.23), p=0.89 | 1.01 (0.83~1.23), p=0.893 |
| Q4 | 0.83 (0.67~1.03), p=0.087 | 0.83 (0.67~1.03), p=0.088 |  | 0.83 (0.67~1.03), p=0.086 | 0.83 (0.67~1.03), p=0.086 |  | 0.83 (0.67~1.03), p=0.086 | 0.83 (0.67~1.03), p=0.086 |  | 0.83 (0.67~1.03), p=0.087 | 0.83 (0.67~1.03), p=0.087 |
| Q5 | 0.97 (0.76~1.25), p=0.832 | 0.97 (0.76~1.25), p=0.84 |  | 0.97 (0.75~1.25), p=0.829 | 0.97 (0.76~1.25), p=0.831 |  | 0.97 (0.76~1.25), p=0.829 | 0.97 (0.76~1.25), p=0.832 |  | 0.97 (0.76~1.25), p=0.834 | 0.97 (0.76~1.25), p=0.836 |
| *T*rend | 0.249 | 0.252 |  | 0.248 | 0.248 |  | 0.248 | 0.248 |  | 0.25 | 0.25 |
| Per SD increment | 0.99 (0.89~1.1), p=0.833 | 0.99 (0.89~1.1), p=0.847 |  | 0.99 (0.89~1.1), p=0.83 | 0.99 (0.89~1.1), p=0.832 |  | 0.99 (0.89~1.1), p=0.831 | 0.99 (0.89~1.1), p=0.833 |  | 0.99 (0.89~1.1), p=0.834 | 0.99 (0.89~1.1), p=0.837 |
|  |  |  |  |  |  |  |  |  |  |  |  |
| Dietary β-carotene intake, mcg/day | |  |  |  |  |  |  |  |  |  |  |
| Q1 | 1(Ref) | 1(Ref) |  | 1(Ref) | 1(Ref) |  | 1(Ref) | 1(Ref) |  | 1(Ref) | 1(Ref) |
| Q2 | 1.07 (0.89~1.29), p=0.477 | 1.07 (0.89~1.29), p=0.477 |  | 1.07 (0.89~1.29), p=0.483 | 1.07 (0.89~1.29), p=0.483 |  | 1.07 (0.89~1.29), p=0.484 | 1.07 (0.89~1.29), p=0.483 |  | 1.07 (0.89~1.29), p=0.478 | 1.07 (0.89~1.29), p=0.48 |
| Q3 | 0.87 (0.71~1.07), p=0.177 | 0.87 (0.71~1.06), p=0.175 |  | 0.87 (0.71~1.06), p=0.176 | 0.87 (0.71~1.06), p=0.173 |  | 0.87 (0.71~1.06), p=0.176 | 0.87 (0.71~1.06), p=0.173 |  | 0.87 (0.71~1.07), p=0.177 | 0.87 (0.71~1.06), p=0.173 |
| Q4 | 1.01 (0.83~1.24), p=0.893 | 1.01 (0.83~1.24), p=0.903 |  | 1.01 (0.83~1.24), p=0.899 | 1.01 (0.83~1.24), p=0.896 |  | 1.01 (0.83~1.24), p=0.899 | 1.01 (0.83~1.24), p=0.896 |  | 1.01 (0.83~1.25), p=0.893 | 1.01 (0.83~1.25), p=0.892 |
| Q5 | 0.84 (0.64~1.1), p=0.197 | 0.84 (0.64~1.09), p=0.189 |  | 0.84 (0.64~1.09), p=0.195 | 0.84 (0.64~1.1), p=0.197 |  | 0.84 (0.64~1.09), p=0.195 | 0.84 (0.64~1.1), p=0.197 |  | 0.84 (0.64~1.1), p=0.198 | 0.84 (0.64~1.1), p=0.198 |
| *T*rend | 0.243 | 0.234 |  | 0.241 | 0.243 |  | 0.242 | 0.244 |  | 0.244 | 0.244 |
| Per SD increment | 0.97 (0.87~1.07), p=0.519 | 0.96 (0.87~1.07), p=0.496 |  | 0.97 (0.87~1.07), p=0.522 | 0.97 (0.87~1.08), p=0.529 |  | 0.97 (0.87~1.07), p=0.522 | 0.97 (0.87~1.08), p=0.529 |  | 0.97 (0.87~1.07), p=0.52 | 0.97 (0.87~1.07), p=0.523 |
| PLCO, prostate, lung, colorectal and ovarian; HR, hazard ratio; CI, confidence interval; SD, standard deviation. | | | | |  |  |  |  |  |  |  |
| Model 3 adjusted for age (continuous) and sex (male vs. female), trial arm (intervention vs. control), race (white, non-Hispanic vs. black, non-Hispanic vs. Hispanic vs. others), marital status (married vs. unmarried), education level (college below vs. college graduate vs. postgraduate), **aspirin use (yes vs. no)**, diabetes (yes vs. no), cigarette smoking (never vs. current vs. former), BMI (<25kg/m2 vs. >=25kg/m2)，family history of colorectal cancer (yes vs. no vs. possibly), energy intake from diet (continuous), alcohol drinking (never vs. former vs. current),supplemental Beta-Carotene (continuous), supplemental vitamin A (continuous), supplemental vitamin E (continuous), supplemental vitamin C (continuous), supplemental calcium (continuous). For the association of dietary α-carotene intake with colorectal cancer incidence, hazard ratios were further adjusted for energy-adjusted dietary β-carotene intake (mcg/day). For the association of dietary β-carotene intake with colorectal cancer incidence, hazard ratios were further adjusted for energy-adjusted dietary α-carotene intake (mcg/day). Model 4 adjusted for all factors of model 3, and further adjusted for some other diseases, including colon comorbidities (yes vs. no), gallbladder stones or inflammation (yes vs. no), heart attack (yes vs. no), colorectal polyps (yes vs. no), stroke (yes vs. no). Model 5 adjusted for all factors of model 3, but excluded aspirin use (yes vs. no).  Model 6 adjusted for all factors of model 4, but excluded aspirin use (yes vs. no). Model 7 adjusted for all factors of model 3, and further adjusted for ibuprofen but not aspirin use. Model 8 adjusted for all factors of model 4, and further adjusted for ibuprofen but not aspirin use. Model 9 adjusted for all factors of model 3, and further adjusted ibuprofen and aspirin use. Model 10 adjusted for all factors of model 4, and further adjusted ibuprofen and aspirin use. | | | | | | | | | | | |

| **Supplementary Table 7**. Additional analyses on the associations between energy-adjusted dietary carrot/carotene intake and colorectal cancer mortality in the PLCO cancer screening trial. | | | | | | | | | | | |  |
| --- | --- | --- | --- | --- | --- | --- | --- | --- | --- | --- | --- | --- |
|  | Hazard ratios (95% confidence interval), P-value | | | | | | | | | | |  |
|  | **only adjusted aspirin** | |  | **not adjusted aspirin** | |  | **only adjusted ibuprofen** | |  | **adjusted ibuprofen and aspirin** | |  |
| Variable | **Model 3** | **Model 4** |  | **Model 5** | **Model 6** |  | **Model 7** | **Model 8** |  | **Model 9** | **Model 10** |  |
|  |  |  |  |  |  |  |  |  |  |  |  |  |
| Dietary carrot intake, g/day | |  |  |  |  |  |  |  |  |  |  |  |
| Q1 | 1(Ref) | 1(Ref) |  | 1(Ref) | 1(Ref) |  | 1(Ref) | 1(Ref) |  | 1(Ref) | 1(Ref) |  |
| Q2 | 0.94 (0.7~1.28), p=0.71 | 0.94 (0.69~1.28), p=0.698 |  | 0.95 (0.7~1.28), p=0.721 | 0.94 (0.69~1.28), p=0.699 |  | 0.95 (0.7~1.28), p=0.722 | 0.94 (0.69~1.28), p=0.7 |  | 0.95 (0.7~1.28), p=0.72 | 0.94 (0.69~1.28), p=0.697 |  |
| Q3 | 0.91 (0.67~1.24), p=0.569 | 0.91 (0.67~1.24), p=0.562 |  | 0.92 (0.67~1.25), p=0.576 | 0.91 (0.67~1.24), p=0.56 |  | 0.92 (0.67~1.25), p=0.577 | 0.91 (0.67~1.24), p=0.56 |  | 0.92 (0.67~1.25), p=0.574 | 0.91 (0.67~1.24), p=0.558 |  |
| Q4 | 0.87 (0.64~1.18), p=0.359 | 0.87 (0.64~1.18), p=0.367 |  | 0.87 (0.64~1.18), p=0.368 | 0.87 (0.64~1.18), p=0.364 |  | 0.87 (0.64~1.18), p=0.368 | 0.87 (0.64~1.18), p=0.364 |  | 0.87 (0.64~1.18), p=0.367 | 0.87 (0.64~1.18), p=0.363 |  |
| Q5 | 0.87 (0.64~1.18), p=0.376 | 0.87 (0.64~1.18), p=0.37 |  | 0.87 (0.64~1.18), p=0.378 | 0.87 (0.64~1.18), p=0.367 |  | 0.87 (0.64~1.18), p=0.377 | 0.87 (0.64~1.18), p=0.366 |  | 0.87 (0.64~1.18), p=0.375 | 0.87 (0.64~1.18), p=0.364 |  |
| *T*rend | 0.297 | 0.3 |  | 0.3 | 0.296 |  | 0.299 | 0.295 |  | 0.297 | 0.294 |  |
| Per SD increment | 0.94 (0.84~1.05), p=0.248 | 0.94 (0.84~1.05), p=0.247 |  | 0.94 (0.84~1.05), p=0.244 | 0.94 (0.84~1.05), p=0.244 |  | 0.94 (0.84~1.05), p=0.244 | 0.94 (0.84~1.05), p=0.244 |  | 0.94 (0.84~1.05), p=0.244 | 0.94 (0.84~1.05), p=0.244 |  |
|  |  |  |  |  |  |  |  |  |  |  |  |  |
| Dietary α-carotene intake, mcg/day | |  |  |  |  |  |  |  |  |  |  |  |
| Q1 | 1(Ref) | 1(Ref) |  | 1(Ref) | 1(Ref) |  | 1(Ref) | 1(Ref) |  | 1(Ref) | 1(Ref) |  |
| Q2 | 0.95 (0.7~1.28), p=0.742 | 0.95 (0.7~1.28), p=0.739 |  | 0.95 (0.7~1.28), p=0.744 | 0.95 (0.7~1.28), p=0.733 |  | 0.95 (0.7~1.28), p=0.741 | 0.95 (0.7~1.28), p=0.731 |  | 0.95 (0.7~1.28), p=0.744 | 0.95 (0.7~1.28), p=0.734 |  |
| Q3 | 0.96 (0.7~1.3), p=0.772 | 0.96 (0.7~1.3), p=0.772 |  | 0.96 (0.7~1.3), p=0.773 | 0.95 (0.7~1.3), p=0.763 |  | 0.96 (0.7~1.3), p=0.772 | 0.95 (0.7~1.3), p=0.762 |  | 0.96 (0.7~1.3), p=0.772 | 0.95 (0.7~1.3), p=0.762 |  |
| Q4 | 0.83 (0.6~1.16), p=0.277 | 0.83 (0.6~1.16), p=0.276 |  | 0.83 (0.6~1.16), p=0.275 | 0.83 (0.6~1.15), p=0.268 |  | 0.83 (0.6~1.16), p=0.273 | 0.83 (0.6~1.15), p=0.267 |  | 0.83 (0.6~1.16), p=0.274 | 0.83 (0.6~1.15), p=0.267 |  |
| Q5 | 0.91 (0.62~1.35), p=0.649 | 0.91 (0.62~1.35), p=0.654 |  | 0.91 (0.62~1.34), p=0.638 | 0.91 (0.62~1.34), p=0.631 |  | 0.91 (0.62~1.34), p=0.636 | 0.91 (0.62~1.34), p=0.629 |  | 0.91 (0.62~1.34), p=0.635 | 0.91 (0.62~1.34), p=0.63 |  |
| *T*rend | 0.417 | 0.42 |  | 0.409 | 0.403 |  | 0.408 | 0.402 |  | 0.407 | 0.401 |  |
| Per SD increment | 0.94 (0.8~1.11), p=0.49 | 0.95 (0.8~1.11), p=0.497 |  | 0.94 (0.8~1.11), p=0.475 | 0.94 (0.8~1.11), p=0.467 |  | 0.94 (0.8~1.11), p=0.473 | 0.94 (0.8~1.11), p=0.467 |  | 0.94 (0.8~1.11), p=0.474 | 0.94 (0.8~1.11), p=0.469 |  |
|  |  |  |  |  |  |  |  |  |  |  |  |  |
| Dietary β-carotene intake, mcg/day | |  |  |  |  |  |  |  |  |  |  |  |
| Q1 | 1(Ref) | 1(Ref) |  | 1(Ref) | 1(Ref) |  | 1(Ref) | 1(Ref) |  | 1(Ref) | 1(Ref) |  |
| Q2 | 1.06 (0.79~1.42), p=0.705 | 1.05 (0.79~1.41), p=0.729 |  | 1.06 (0.79~1.42), p=0.701 | 1.05 (0.79~1.41), p=0.724 |  | 1.06 (0.79~1.42), p=0.699 | 1.05 (0.79~1.41), p=0.723 |  | 1.06 (0.79~1.42), p=0.698 | 1.05 (0.79~1.41), p=0.724 |  |
| Q3 | 0.82 (0.6~1.13), p=0.225 | 0.82 (0.59~1.13), p=0.219 |  | 0.82 (0.6~1.13), p=0.23 | 0.82 (0.6~1.13), p=0.222 |  | 0.82 (0.6~1.13), p=0.231 | 0.82 (0.6~1.13), p=0.222 |  | 0.82 (0.6~1.13), p=0.231 | 0.82 (0.6~1.13), p=0.222 |  |
| Q4 | 0.9 (0.65~1.25), p=0.521 | 0.9 (0.65~1.24), p=0.507 |  | 0.9 (0.65~1.25), p=0.528 | 0.9 (0.65~1.24), p=0.52 |  | 0.9 (0.65~1.25), p=0.529 | 0.9 (0.65~1.24), p=0.519 |  | 0.9 (0.65~1.25), p=0.528 | 0.9 (0.65~1.24), p=0.518 |  |
| Q5 | 0.91 (0.61~1.36), p=0.64 | 0.9 (0.6~1.35), p=0.619 |  | 0.91 (0.61~1.36), p=0.646 | 0.91 (0.61~1.36), p=0.647 |  | 0.91 (0.61~1.36), p=0.646 | 0.91 (0.61~1.36), p=0.646 |  | 0.91 (0.61~1.36), p=0.647 | 0.91 (0.61~1.36), p=0.643 |  |
| *T*rend | 0.344 | 0.332 |  | 0.352 | 0.352 |  | 0.352 | 0.352 |  | 0.352 | 0.35 |  |
| Per SD increment | 1.00 (0.86~1.17), p=0.959 | 1 (0.86~1.17), p=0.978 |  | 1 (0.86~1.17), p=0.955 | 1.01 (0.86~1.17), p=0.939 |  | 1 (0.86~1.17), p=0.955 | 1.01 (0.86~1.17), p=0.94 |  | 1 (0.86~1.17), p=0.955 | 1.01 (0.86~1.17), p=0.945 |  |
| PLCO, prostate, lung, colorectal and ovarian; HR, hazard ratio; CI, confidence interval; SD, standard deviation.  Model 3 adjusted for age (continuous) and sex (male vs. female), trial arm (intervention vs. control), race (white, non-Hispanic vs. black, non-Hispanic vs. Hispanic vs. others), marital status (married vs. unmarried), education level (college below vs. college graduate vs. postgraduate), aspirin use (yes vs. no), diabetes (yes vs. no), cigarette smoking (never vs. current vs. former), BMI (<25kg/m2 vs. >=25kg/m2)，family history of colorectal cancer (yes vs. no vs. possibly), energy intake from diet (continuous), alcohol drinking (never vs. former vs. current),supplemental Beta-Carotene (continuous), supplemental vitamin A (continuous), supplemental vitamin E (continuous), supplemental vitamin C (continuous), supplemental calcium (continuous). For the association of dietary α-carotene intake with colorectal cancer mortality, hazard ratios were further adjusted for energy-adjusted dietary β-carotene intake (mcg/day). For the association of dietary β-carotene intake with colorectal cancer mortality, hazard ratios were further adjusted for energy-adjusted dietary α-carotene intake (mcg/day). Model 4 adjusted for all factors of model 3, and further adjusted for some other diseases, including colon comorbidities (yes vs. no), gallbladder stones or inflammation (yes vs. no), heart attack (yes vs. no), colorectal polyps (yes vs. no), stroke (yes vs. no). Model 5 adjusted for all factors of model 3, but excluded aspirin use (yes vs. no).  Model 6 adjusted for all factors of model 4, but excluded aspirin use (yes vs. no). Model 7 adjusted for all factors of model 3, and further adjusted for ibuprofen but not aspirin use. Model 8 adjusted for all factors of model 4, and further adjusted for ibuprofen but not aspirin use. Model 9 adjusted for all factors of model 3, and further adjusted ibuprofen and aspirin use. Model 10 adjusted for all factors of model 4, and further adjusted ibuprofen and aspirin use. | | | | | | | | | | | |  |
|  |  |  |  |  |  |  |  |  |  |  |  |  |
|  |  |  |  |  |  |  |  |  |  |  |  |  |
|  |  |  |  |  |  |  |  |  |  |  |  |  |
|  |  |  |  |  |  |  |  |  |  |  |  |  |
|  |  |  |  |  |  |  |  |  |  |  |  |  |
|  |  |  |  |  |  |  |  |  |  |  |  |  |
|  |  |  |  |  |  |  |  |  |  |  |  |  |
|  |  |  |  |  |  |  |  |  |  |  |  |  |
|  |  |  |  |  |  |  |  |  |  |  |  |  |
|  |  |  |  |  |  |  |  |  |  |  |  |  |
|  |  |  |  |  |  |  |  |  |  |  |  |  |
|  |  |  |  |  |  |  |  |  |  |  |  |  |
|  |  |  |  |  |  |  |  |  |  |  |  |  |
|  |  |  |  |  |  |  |  |  |  |  |  |  |
